# Genome‐wide transcriptomic changes reveal the genetic pathways involved in insect migration

**DOI:** 10.1111/mec.16588

**Published:** 2022-07-12

**Authors:** Toby Doyle, Eva Jimenez‐Guri, Will L. S. Hawkes, Richard Massy, Federica Mantica, Jon Permanyer, Luca Cozzuto, Toni Hermoso Pulido, Tobias Baril, Alex Hayward, Manuel Irimia, Jason W. Chapman, Chris Bass, Karl R. Wotton

**Affiliations:** ^1^ Centre for Ecology and Conservation University of Exeter, Cornwall Campus Penryn UK; ^2^ Centre for Genomic Regulation Barcelona Institute of Science and Technology Barcelona Spain; ^3^ Universitat Pompeu Fabra Barcelona Spain; ^4^ ICREA Barcelona Spain; ^5^ Environment and Sustainability Institute University of Exeter, Cornwall Campus Penryn UK; ^6^ Department of Entomology, College of Plant Protection Nanjing Agricultural University Nanjing People's Republic of China

**Keywords:** differential gene expression, genetics of migration, insect migration, migratory hoverflies, molecular adaptations, syrphidae

## Abstract

Insects are capable of extraordinary feats of long‐distance movement that have profound impacts on the function of terrestrial ecosystems. The ability to undertake these movements arose multiple times through the evolution of a suite of traits that make up the migratory syndrome, however the underlying genetic pathways involved remain poorly understood. Migratory hoverflies (Diptera: Syrphidae) are an emerging model group for studies of migration. They undertake seasonal movements in huge numbers across large parts of the globe and are important pollinators, biological control agents and decomposers. Here, we assembled a high‐quality draft genome of the marmalade hoverfly (*Episyrphus balteatus*). We leveraged this genomic resource to undertake a genome‐wide transcriptomic comparison of actively migrating *Episyrphus*, captured from a high mountain pass as they flew south to overwinter, with the transcriptomes of summer forms which were non‐migratory. We identified 1543 genes with very strong evidence for differential expression. Interrogation of this gene set reveals a remarkable range of roles in metabolism, muscle structure and function, hormonal regulation, immunity, stress resistance, flight and feeding behaviour, longevity, reproductive diapause and sensory perception. These features of the migrant phenotype have arisen by the integration and modification of pathways such as insulin signalling for diapause and longevity, JAK/SAT for immunity, and those leading to octopamine production and fuelling to boost flight capabilities. Our results provide a powerful genomic resource for future research, and paint a comprehensive picture of global expression changes in an actively migrating insect, identifying key genomic components involved in this important life‐history strategy.

## 1 INTRODUCTION

Migration is a diverse and widespread phenomenon, occurring throughout the animal kingdom. The traits needed to sustain periods of long‐distance movement, characteristic of many migrants, are wide ranging and include morphological, physiological and behavioural adaptations that evolved together to form the migratory syndrome (Dingle, 2006). It is now well established that phenotypic variation seen in migrants has a genetic basis (Liedvogel et al., 2011; Merlin & Liedvogel, 2019) and since the first strands of evidence from selective breeding experiments in birds, increasingly sophisticated molecular techniques and computing power have allowed the role of genetics in migration to be explored in a variety of taxa (Berthold & Querner, 1981; Delmore et al., 2020, 2015; Helbig, 1991, 1992, 1996; Helbig et al., 2010; Jones et al., 2015, 2008; Wang et al., 2014; Zhan et al., 2014).

Insects are the most numerous terrestrial migrants, far surpassing vertebrates in terms of biomass and abundance, and having profound impacts on ecosystem processes (Chapman et al., 2015; Hu et al., 2016; Satterfield et al., 2020). In addition, insects are well suited to experimental and genetic studies and have been used to investigate the genetics of migratory traits. In the monarch butterfly (*Danaus plexippus*), Zhan et al. (2014) identified genomic regions separating migratory North American populations from others that had independently lost migration, revealing a total of 5.14 Mb (2.1%) of the genome, including 690 predicted genes which were associated with migration (Zhan et al., 2014). In contrast, Jones et al. (2015) used transcriptomic analysis of migratory prereproductive cotton bollworms (*Helicoverpa armigera*), comparing short‐ and long‐distance fliers from the same populations in Greece and among populations in China (Jones et al., 2015). In total 215 candidate genes for long‐distance flight where identified, the majority in populations from China, including genes important for the mobilisation and synthesis of lipids as flight fuel, the development of flight muscle structure and the regulation of hormones that influence migratory physiology (Jones et al., 2015). Finally, the migratory locust (*Locusta migratoria*) has long been a model for many aspects related to phase change—the switch between solitary and gregarious forms—and for long distance flight, in studies utilising both genomic and transcriptomic approaches (Chen et al., 2010; Wang et al., 2014).

A great diversity of other insects display migratory life‐histories, offering an opportunity to develop a comparative framework to elucidate the molecular underpinnings and evolution of migration. An emerging model group for the study of migration are hoverflies, which compose the dipteran family Syrphidae. Hoverflies include a large number of migratory species that undertake migrations on every continent except Antarctica (Aubert et al., 1976; Beebe, 1949; Finch & Cook, 2020; Hill, 2013; Jia et al., 2022; Menz et al., 2019; Shannon, 1926; Westmacott & Williams, 1954; Wotton et al., 2019). Migration has arisen multiple times within this family and is found within both the Syrphinae and Eristalinae subfamilies. Species within these groups are of huge economic importance through the pollination of crops and wildflowers, decomposition of organic matter and pest regulation services they provide, and they can be extremely numerous (Doyle et al., 2020; Wotton et al., 2019). In addition, their relatively close relatedness to the fruit fly, *Drosophila melanogaster*, assists in orthology assignment for the prediction of gene function, while numerous genomes are available or in production which will greatly aid in future investigations into the molecular evolution of migratory traits (Hawkes & Wotton, 2021, 2022). Importantly, various hoverfly species can also be cultured in the lab and protocols and transgenic lines have been established to investigate gene function, making them a promising model to study the genetics of migration (Lemke et al., 2010; Wang et al., 2022).

**FIGURE 1:**
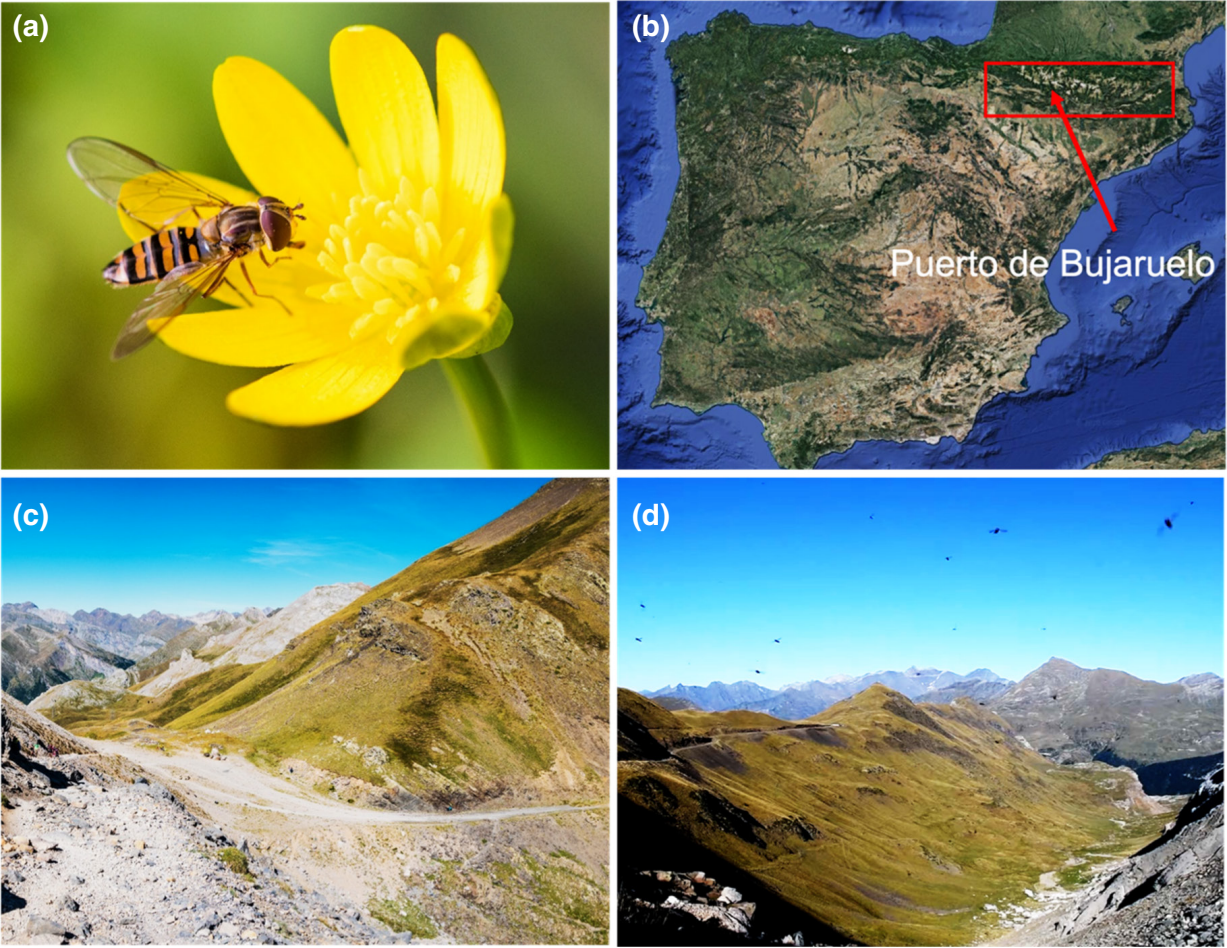
Marmalade hoverfly migration. (a) A female marmalade hovefly, *Episyrphus balteatus*, feeding on a buttercup. (b) Location of the mountain pass of Bujaruelo (Puerto de Bujaruelo; red arrow), a 2273 m pass on the French–Spanish border in the Pyrenees (red box). Image from Google Maps. (c) The 30 m wide pass of Bujaruelo concentrates migrants crossing the Pyrenees. (d) Active *Episyrphus* migration (black dots on skyline) over the pass in October

A key study species within the migratory hoverflies is the marmalade hoverfly *Episyrphus balteatus* (De Geer, 1776)—hereafter referred to as *Episyrphus* (Figure 1a). *Episyrphus* belongs to the tribe Syrphini, and undertakes seasonal migration, with individuals travelling thousands of kilometres in the autumn from northern Europe to southern Europe and North Africa to overwinter (Sahib et al., 2020; Wotton et al., 2019). Populations emerging in the spring undergo a multigenerational migration back northwards, repopulating seasonally productive latitudes. This pattern is supported by isotopic analysis, numerous observations and by radar studies that have revealed seasonally beneficial directions of movement: north in spring (May–June) and south in autumn (August–September; Aubert et al., 1976; Gatter & Schmid, 1990; Lack & Lack, 1951; Ouin et al., 2011; Owen, 1956; Wotton et al., 2019). In contrast, summer *Episyrphus* display no strong group orientation, corresponding with a period of plentiful resources, high reproductive rates and lower mobility (Wotton et al., 2019). Examination of genetic markers from *Episyrphus* across Europe has revealed a high level of genetic mixing and a lack of isolation by distance, confirming North–South migratory movements and indicating a connected population (Hondelmann et al., 2005; Raymond et al., 2013). Extensive genetic mixing has also been seen in *Episyrphus* populations in China (Jia et al., 2022). Migration appears to be achieved by a combination of high‐altitude wind‐assisted flight and, when facing headwinds, low‐level flight within the flight boundary layer (FBL; Gao et al., 2020; Lack & Lack, 1951; Wotton et al., 2019). Flying within the FBL lessens the impact of headwinds with migration often observed along coasts or through mountain passes where topology serves to concentrate migrant number, for example in the mountain pass of Bujaruelo in the Pyrenees (Figure 1b–d). This feature of hoverfly migration allows the collection and study of individuals actively expressing the migratory syndrome (Massy et al., 2021).

The mechanisms that enable hoverflies to undertake long‐distance migrations are poorly understood; however, as in other insect migrants, autumn *Episyrphus* are in a state of reproductive diapause, store additional fat and show increased longevity (Hondelmann & Poehling, 2007). In addition, while males begin the migration along with the females, the population becomes dominated by mated females as migration proceeds, leaving only the females to overwinter (Hondelmann & Poehling, 2007; Rotheray & Gilbert, 2011). The behaviour of migrant *Episyrphus* observed in the mountain pass of Bujaruelo is very different to that seen in summer forms, with individuals displaying a focused directional movement undistracted by external cues such as floral resources (KRW personal observation). Ground speeds of migratory flight have been measured at 3–5 m/s (12–18 km/h) through the mountains and 10 m/s (36 km/h) at high altitude (Aubert et al., 1969; Gao et al., 2020). As migration may occur over many hours, and as hoverflies are not capable of soaring flight as seen in some butterfly and dragonfly migrants, significant energy must be required for these journeys.

Another trait seen in migrants is their ability to orientate in seasonally beneficial directions and migratory hoverflies have recently been shown to use an innate time‐compensated sun compass and to partially compensate for wind drift (Gao et al., 2020; Massy et al., 2021). Surprisingly, despite these differences between migratory and summer hoverflies, investigations into migrant morphology using wing morphometrics have so far revealed little change to non‐migratory stages, leaving the full extent of the underlying differences between forms an open question (Raymond et al., 2014). To isolate potential differences that may not be morphologically apparent, we utilise genome sequencing coupled with RNA‐seq analysis of actively migrating and nonmigratory summer hoverflies, to identify differentially expressed genes associated with migration.

## 2 MATERIALS AND METHODS

### 2.1 Genome sequencing

High molecular weight DNA was extracted from two female *Episyrphus* (KatzBiotech strain). DNA extraction was performed using an adapted CTAB/phenol protocol outlined by the Joint Genome Institute. Resulting DNA quality and quantity were assessed using Qubit BR dsDNA quantification kit (Invitrogen), Nanodrop (Invitrogen) and Bioanalyser 2100 (Agilent). Two samples were pooled and sent for PacBio library construction with Exeter Sequencing Service following Pacific Biosciences guidelines for gDNA libraries. In short, this consisted of 100 ng of DNA ran on a pulsed field gel electrophoresis to estimate molecular weight (BioRad CHEF‐DR III). The sample was prepared using the SMRTbell express template preparation kit 2.0 without the DNA shearing steps. Subsequently the library was size selected on the BluePippin High‐Pass Plus gel cassette following the manufacturer's guidelines for DNA >15 kb. This library was prepared for sequencing on the PacBio Sequel using chemistry 2.1 at a loading density of 6 pmol and sequenced on 4 SMRT cells.

### 2.2 Genome assembly

PacBio reads were assembled using canu 1.8 (Koren et al., 2017) using a corrected error rate of 0.105 followed by running the Purge Haplotigs (Roach et al., 2018) tool for detecting and removing the haplotigs generated during the assembly step. Raw PacBio reads were mapped to each assembly using minimap2 (Li, 2018) and the resulting alignments used for haplotig detection and removal. To further improve assembly contiguity, we trimmed RNA‐seq reads obtained by Illumina sequencing (see transcriptome sequencing section) using skewer (Jiang et al., 2014) and mapped them to the assembled genomes using the splicing aware aligner STAR (Dobin et al., 2013). STAR was run allowing the detection of chimeric reads (i.e., reads able to align to different contigs), we used this information for detecting candidate sequences that were condensed into larger portions (scaffolds). STAR alignments were fed to rascaf to produce scaffolds (Song et al., 2016). The genome assembly was evaluated for completeness by inspecting the detection of conserved genes using the benchmarking universal single‐copy orthologues (BUSCO) pipeline (Seppey et al., 2019; Table 1).

### 2.3 Genome annotation

Known repeats were masked on the assembly using RepeatMasker (Smit et al., 2013; Tarailo‐Graovac & Chen, 2009) with the Dfam database (Hubley et al., 2016; selecting repeats from Diptera). RNAseq data (see transcriptome sequencing section) was mapped onto the reference genome to generate an assembled transcriptome using Stringtie (Pertea et al., 2015). Both the masked genome and the assembled transcriptome were then used by Augustus (Stanke & Morgenstern, 2005) to generate gene annotations (Table 1), yielding 11,616 protein‐coding genes. To complete this annotation, we ran Transdecoder version 2.0.1 (PMID: 23845962) on the Stringtie transcripts generated from the RNAseq data and added those that were predicted to encode proteins and that did not share exons with Augustus gene models, yielding 2932 additional gene models. This step increased the percent of protein‐coding genes with a *D. melanogaster* blast hit from 76.5% to 80.3%. Additional assignment of orthology was carried out using EGG‐NOG mapper (Cantalapiedra et al., 2021). A full transposable element (TE) annotation and quantification was carried out using the Earl Grey TE annotation pipeline with both the RepBase (version 23.08) and Dfam (version 3.4) TE sequence databases (Baril et al., 2021; Hubley et al., 2016; Jurka et al., 2005), revealing a total repeat content of 52%. Best hit BLAST searches were run against *D. melanogaster* and human gene sets to assign putative gene names. Finally, the genome was also inspected for the presence of noncoding genes using the cmsearch tool from the infernal suite (Nawrocki, 2014) with reference to the Rfam database (Griffiths‐Jones, 2003), resulting in 1855 predicted noncoding RNAs (including 623 tRNAs and 899 microRNAs). A full genome report and BUSCO scores are provided in Tables S1 and S2. Protein‐coding gene models from Augustus were named EBAG0, non‐coding RNA gene models EBAG1 and Stringtie based ones EBAG2.

### 2.4 Transcriptome sequencing and expression analysis

Female migrant flies were collected during active migration over the Pyrenean pass of Puerto de Bujaruelo, Spain (42.7038793 N, −0.0641454 W; Figure 1) during September–October 2018, while nonmigrant samples were collected from females during summer (13–18 July 2019) from the University of Exeter campus in Penryn, Cornwall. Collection data are provided in Table S3. These samples are all drawn from a single European panmictic population. All samples were snap frozen in the field using a dry shipper and stored at −80°C. Total RNA was extracted using standard TRIzol protocol (Invitrogen) and treated with DNAse using TURBO DNAse (Invitrogen) following the manufacturer's guidelines. Quality and quantity of total RNA was assessed using Qubit BR RNA quantification kit (Invitrogen), Nanodrop (Invitrogen) and Bioanalyser 2100 (Agilent).

**FIGURE 2:**
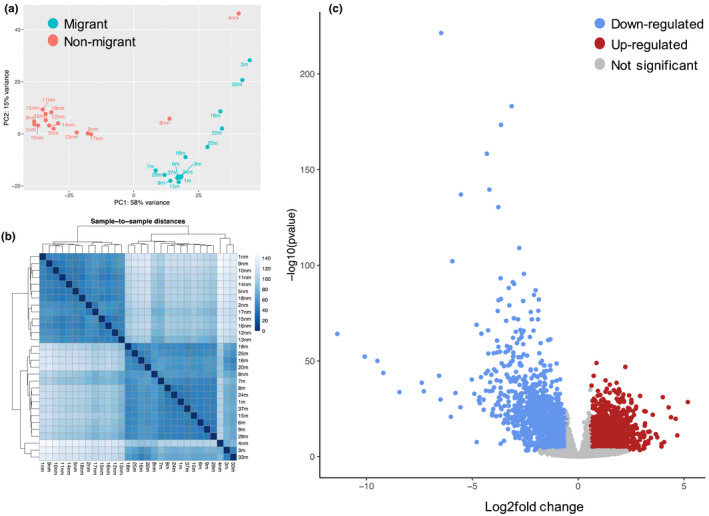
Sample similarities and differential gene expression. (a) Principal component plot of the samples from nonmigrants (pink) and migrant (turquoise). (b) A heat map of the sample‐to‐sample distances and clustering of the samples. (c) Volcano plot of downregulated (blue), upregulated (red) and nonsignificant (grey) genes at a log2fold change of 1.5 and an adjusted *p*‐values of <.001

In total, 30 RNA samples (15 migrant and 15 nonmigrant) were sent to Edinburgh Genomics for library construction and RNA‐seq. TruSeq stranded mRNA‐seq libraries were prepared from total RNA and sequences generated on the NovaSeq platform using 50 base pair (bp) pair‐end (PE) sequencing. Reads were mapped to the reference genome using STAR to produce a list of read counts per gene. The list was fed to DESeq2 (Love et al., 2014) for estimating differentially expressed genes (DGE), comparing migrant to nonmigrant individuals (Supporting Information File S2). The read counts were log transformed and variance stabilized using the DESeq2 function vst before being used for calculating a distance matrix among the samples and a principal component analysis (PCA; Figure 2). Gene expression changes where further visualised using morpheus (https://software.broadinstitute.org/morpheus).

In addition, total RNA from pooled tissue samples of laboratory reared *Episyrphus* (ovary, brain, testis, muscle, fat body, malpighian tubes, epidermis and gut) was obtained through the following steps. Dissected tissues were kept in cold RNAlater (Invitrogen AM7024) until RNA extraction. To homogenize the samples, tissues were rinsed with sterile PBS and put in tubes with 500 μl of Trizol (Ambion 15,596,018) and some glass beads (Sigma G8772) and homogenized for 45 s in a Minibeadbeater (Biospec Products). Tissue‐TRIzol mix was transferred to Phasemaker tubes (Thermofisher A33248) to facilitate the clean recovery of the inorganic phase. The purification of the RNA in the inorganic phase was done following manufacturer's instructions. Extracted RNA was resuspended in 11 μl of RNAse free water and quantified using Qubit RNA Assay kit (Invitrogen Q32855). Illumina polyA‐selected RNA‐seq libraries were prepared and sequenced in a HiSeq2500 machine to generate an average of ~75 million 125‐nt paired‐end reads. Rearing conditions for laboratory *Episyrphus* were 21°C day and 15°C night at 80% humidity with a 16.40:7.20 L:D cycle. We pooled tissues from 10 individuals for samples from brain, muscle, gut, epidermis and malpighian tubes, eight from developed and three from immature ovaries, 20 from the testes and six from the larval fat body.

### 2.5 Quantitative PCR (qPCR)

Reference gene primers were designed by blasting *D. melanogaster* reference genes against the *Episyrphus* genome (Ponton et al., 2011) with the resulting hits verified through reciprocal blasts. Short fragment primers were generated using Primer3 (Untergasser et al., 2012). Genes of interest (GOI) primers were based on outputs from DGE analysis and designed in the same way as reference primers. Reference genes were analysed in a GeNorm pilot study to determine gene stability using the software Qbase+ (Biogazelle), resulting in two genes being selected for normalisation. Primer combinations for GOI and reference genes can be found in Table S3. Primer efficiency was calculated using a serial dilution of experimental cDNA in 10‐ or 5‐fold dilutions over a minimum of 5 points. An efficient reaction was deemed to be between 90% and 110%, our reference and GOI primers achieved efficiencies from 94% to 105% (Broeders et al., 2014; Taylor et al., 2010). To generate cDNA libraries reverse transcription was performed using GoScript reverse transcriptase (Promega) following the manufacturer's guidelines with 450 ng of total RNA, random hexamer (50 μM) and oligo dt (16) (50 μM), cDNA was diluted with nuclease free water to 2.25 ng/μl based on RNA starting quantity. Quantitative PCR (qPCR) was carried out using 4 ul of cDNA (9 ng) in 20 μl reactions containing 2× SensiFAST SYBR Hi‐ROX kit (Bioline) and 0.6 μM (0.6 μl of 10 μM stock) of each primer. Five replicates from sequenced individuals were used for migrants and nonmigrants, these were run in duplicates on Primer Design BrightWhite qPCR plates. Samples were run on an Applied Biosystem's Step One plus qPCR machine using the following cycling program 95°C for 2 min, followed by 40 cycles of 95°C for 5 s, 60°C for 10 s and 72°C for 15 s, then a melt curve analysis from 60 to 95°C. Relative expression of migration‐associated genes within migratory samples was compared to controls (verified nonmigratory samples), these where normalised using the geometric mean of two stable reference genes as described in (Hellemans et al., 2007; Vandesompele et al., 2002). Gene maximisation plate set up was applied as only five biological replicates from control and experimental samples were run allowing for all samples of a GOI to be run in one go with reference primers. Reference primers were used in each subsequent plate along with negative template and reserve transcription controls. All other qPCR parameter were set up according to the MIQE best practise guidelines (Bustin et al., 2009). Expression differences and validation can be found in Table S5 and Figures S1 and S2.

### 2.6 GO enrichment analysis

Gene ontology (GO) enrichment analysis was carried out in GOrilla (Eden et al., 2009) to identify overrepresented GO terms from up‐ and downregulated gene sets compared to all expressed genes from migrants and nonmigrants (Tables S7 and S8). Significant GO terms were mapped using the Revigo tool to remove redundant terms and visualise the semantic similarity of the remaining terms (Supek et al., 2011).

## 3 RESULTS

### 3.1 Genome assembly and annotation

We present a draft genome assembly produced using 41 Gb of PacBio long reads from two pooled females, and 72 Gb of expression data using RNA‐seq of whole bodies and multiple tissues. The resulting genome assembly is 568 Mb in size with a scaffold N50 of 3.3 Mb and a contig number of 1908 (see assembly and annotations statistics in Table 1 and Table S1). Assessment for completeness using a Diptera BUSCO data set (diptera_odb10: 3285 genes) reached 94% for compete BUSCO genes (Table 1 and Table S2). Genome annotation using prediction and transcript evidence resulted in a total of 14,548 protein‐coding genes. Of these 10,479 were assigned orthologs using EGG‐NOG mapper, including 8780 showing 1:1 orthology to genes in *D. melanogaster*. Total repeat content was 52%.

**TABLE 1:** Assembly features table for the genome of the marmalade hoverfly *Episyrphus balteatus*

Bioproject	PRJNA720810
Assembly identifier	JAGTYB000000000
Genome size (Mb)	565 Mb
N50	2.5 Mb
BUSCO genome score (Diptera data set)	C: 93.7% [S: 90.6%, D: 3.1%], F: 2.3%,M: 4.0%, *n*: 3285
Number of protein‐coding genes	14,548
Contigs (>500 bp)	1231
Largest contig	13.9 Mb
Repeat content	DNA: 13.12%, Rolling Circle: 3.47%, Penelope‐like: 0.38%, LINE: 4.64%, SINE: 0.10%, LTR: 3.99%, Unclassified: 25.88%, Other (simple repeat): 0.76%

*Note*: BUSCO scores based on the diptera_odb10 BUSCO set using version 4.1.2. A full report and set of BUSCO scores are available in Tables S1 and S2.

Abbreviations: C, complete (S, single copy, D, duplicated); F, fragmented; M, missing; *n*, number of orthologues in comparison.

### 3.2 Identification of migration‐associated genes by transcriptomics

To identify transcripts involved in long‐distance migration we compared the genome‐wide transcription profiles of actively migrating individuals, caught as they traversed a pass in the Pyrenees on their journey south, with summer individuals originating from the UK (Figure 1a–d). Total RNA was extracted from 15 whole individuals of each group and subject to Illumina cDNA library preparation and sequencing. A total of 1438 million paired‐end 50‐bp reads were sequenced with an average yield of 48 million reads per sample. Following quality control, reads were aligned to the annotated *Episyrphus* genome using STAR to produce a list of read counts per gene. Differential gene expression was estimated using DESeq2. PCA analysis showed distinct signatures for migrant and summer forms, however two nonmigrants (4 and 8 nm) displayed intermediate signatures (Figure 2a) and a heatmap distance matrix confirmed the overall similarities (Figure 2b). A total of 1543 genes out of an estimated total number of 14,548 genes present in the genome (i.e., 10.6%) showed very strong evidence of differential expression, using an adjusted *p*‐value of <.001 and a log2fold change of 1.5 or more in expression levels. Of the differentially expressed genes, 789 (51%) were upregulated and 754 (49%) were downregulated (Figure 2c; Supporting Information File S2). To validate our expression data, quantitative PCR (qPCR) was used to measure expression levels from a subset of 10 differentially expressed genes: five upregulated, five downregulated (Table S4). We detected a strong correlation between observed fold changes between the RNA‐seq and qPCR data (*R* ^2^ = 0.88) indicating good agreement between both datasets (Table S5, Figure S1).

### 3.3 differentially expressed genes

We examined the top 20 up‐ and downregulated genes ordered by adjusted p‐value and fold change in expression levels and vice versa—that is, differentially expressed genes with the highest significance scores or the highest fold expression changes. We identified the putative roles of these genes using data primarily from *D. melanogaster* orthologues. Over half of the top upregulated genes are associated with metabolic processes (12/40), with other processes including cuticle function (6/40), muscle function (5/40), hormonal processes (4/40), sensory functions (2/40), circadian function, detoxification and signal transduction (Table 2). In contrast, the top downregulated genes include loci predominantly involved in egg formation and oogenesis but also genes related to insulin and TGF‐β signalling, hormonal regulation and fat accumulation (Table S6).

**TABLE 2:** Top upregulated genes. Lists are ordered by adjusted *p*‐value (padj) and log2fold expression differences

Gene ID	Gene symbol	Fold	padj	Role
Top by padj				
EBAG0004479	*LamC*	4.97	1.11E‐45	Muscle function
EBAG0002598	*Cyp4s3*	10.96	4.67E‐44	Detoxification
EBAG0009494	*pdgy*	3.29	1.21E‐37	Metabolic processes
EBAG0002368	*Tret1‐1*	3.77	7.11E‐35	Metabolic processes
EBAG0010159	*vri*	3.72	3.18E‐34	Circadian function
EBAG0010245	*CG6428 (Asparaginase)*	2.84	8.84E‐34	Metabolic processes
EBAG0009869	*jv*	3.82	2.94E‐33	Muscle function
EBAG0001452	*Pde11*	3.18	3.50E‐33	Signal transduction
EBAG0000995	*alpha‐Est9*	3.57	4.68E‐32	Detoxification
EBAG0005848	*Lsp1gamma*	21.06	3.78E‐31	Muscle function
EBAG0009128	*Obp56a*	5.8	4.85E‐31	Sensory functions
EBAG0000239	*Desat1*	8.58	6.95E‐31	Metabolic processes
EBAG2000452	*Nep1*	4.53	1.21E‐30	Sensory functions
EBAG0008744	*Men*	4.17	1.31E‐29	Metabolic processes
EBAG2000084	*Hex‐A*	3.32	2.45E‐29	Metabolic processes
EBAG0007428	*CG11852 (Takeout superfamily)*	4.41	2.95E‐29	Hormonal processes
EBAG0000112	*GstD1*	6.34	5.74E‐29	Detoxification
EBAG0001965	*CG2680 (HAD family)*	3.77	6.14E‐29	Hormonal processes
EBAG2002106	*CG14687*	4.71	2.69E‐28	Muscle function
EBAG0002152	*CG6409*	3.56	4.44E‐28	Unknown
Top by log2fold				
EBAG0000970	*CG8132 (Omega‐amidase)*	26.93	1.06E‐27	Metabolic processes
EBAG2001904	*CG4830 (Long‐chain‐fatty‐acid‐CoA ligase)*	22.22	2.76E‐11	Metabolic processes
EBAG0003336	*CG17191 (Triacylglycerol lipase)*	21.33	1.67E‐19	Metabolic processes
EBAG0005851	*Lsp1alpha*	20.08	5.49E‐22	Muscle function
EBAG0004000	*CG9522*	18.89	1.12E‐11	Hormonal processes
EBAG0003101	*Act88F*	18.47	3.51E‐08	Muscle function
EBAG0011563	*CG17292*	18.4	8.31E‐08	Metabolic processes
EBAG0004744	*Ance‐4*	18.33	4.61E‐25	Unknown
EBAG0008715	*CG7203 (CPLCG cuticle family)*	16.75	7.74E‐08	Cuticle function
EBAG0006977	*Cpr65Av*	16.08	1.10E‐05	Cuticle function
EBAG0001086	*Acp1 (CPLCG cuticle family)*	15.8	5.82E‐07	Cuticle function
EBAG2001604	*CG7214 (CPLCG cuticle family)*	14.1	3.05E‐10	Cuticle function
EBAG0003070	*Cpr100A*	14.01	1.90E‐08	Cuticle function
EBAG0008969	*CG14476 (GCS2α)*	13.52	9.86E‐12	Metabolic processes
EBAG0005758	*Mal‐A7*	13.11	1.27E‐12	Metabolic processes
EBAG2001784	*fiz (CG9509)*	12.45	1.90E‐21	Hormonal processes
EBAG0004866	*CG5390*	11.89	4.51E‐09	Sensory functions
EBAG0006741	CG30101 (Vajk4)	11.50	6.05E‐10	Cuticle function
EBAG0008368	*CG11099*	11.5	2.96E‐12	Unknown
EBAG0000130	*Rootletin*	11.48	3.89E‐13	Sensory functions

*Note*: See Table S6 for downregulated list.

### 3.4 Functional gene enrichment analysis

**FIGURE 3:**
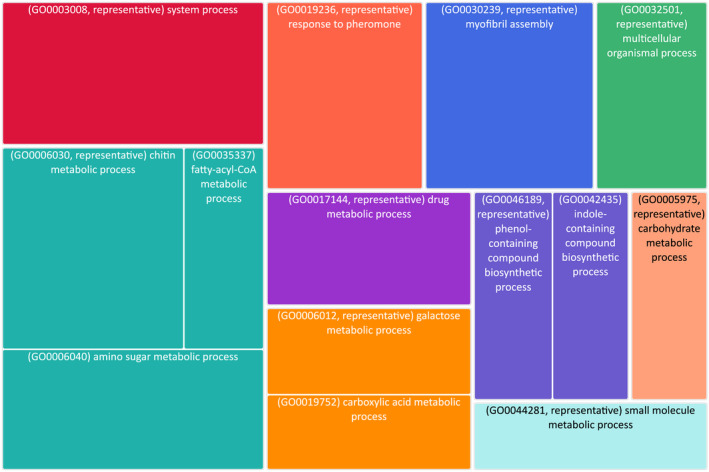
TreeMap of over represented GO process terms in the upregulated gene set. Each rectangle is a single cluster representative and is joined into “superclusters” of loosely related terms, visualized with different colours. The size of the rectangles reflects the enrichment *p*‐values

To further categorise the up‐ and downregulated set of genes, we performed a gene ontology (GO) enrichment analysis using the GOrilla tool followed by Revigo to remove redundant terms and summarise GO terms (Eden et al., 2009; Supek et al., 2011; Figure 3; Tables S7 and S8). For the complete upregulated gene set we found the most significant enrichment in the system process GO term, which includes the representative terms of sensory perception, sensory perception of smell, sensory perception of chemical stimulus and nervous system processes. Other enriched GO terms included response to pheromone, myofibril assembly, chitin metabolic processes, fatty‐acyl‐coA metabolic processes, phenoyl‐containing compound biosynthetic process and carbohydrate metabolic process (see Figure 3 and Table S7 for full list). For the complete downregulated gene set we found a large set of enrichment for GO terms associated with active reproduction including cell cycle processes, reproductive processes and DNA metabolic processes (see Table S8).

## 4 DISCUSSION

**FIGURE 4:**
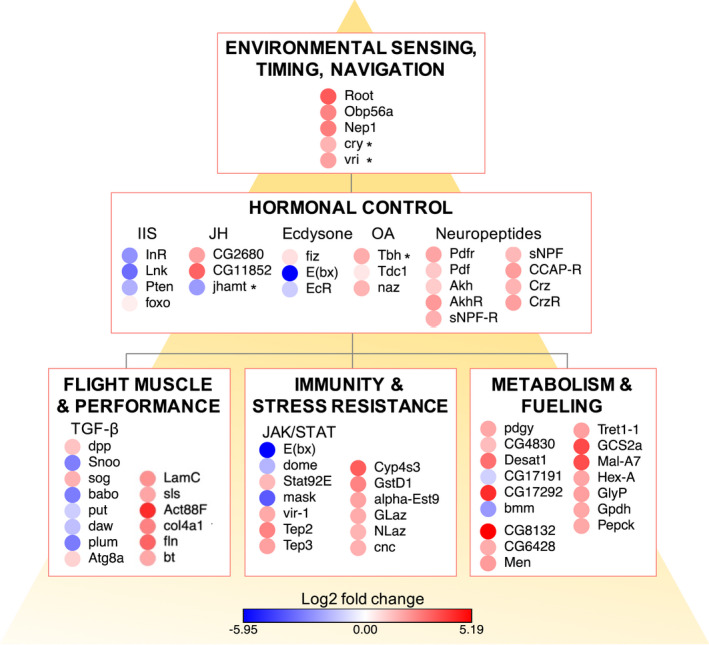
Differential gene expression in migrant *Episyrphus*. Genes appearing in the discussion and their assignment to functional categories along with expression calculated as log2fold change. Gene are placed within a theoretical framework for the genetic basis of migration depicting a cascade from environmental sensing, through hormonal control to down‐stream changes in migratory traits. Top: Environmental sensing of cues such as photoperiod, mediated by photosensitive proteins such as *cry*, modify circadian control of gene expression. IIS and circadian regulation converge on *vri* to modulate behavioural rhythms associated with migration such as flight and feeding. Middle: Hormonal control through IIS, JH, ecdysone pathways control reproductive diapause and longevity while also increasing energy stores and stress tolerance. Upregulation of octopamine (OA) synthesis pathways supply the high OA concentrations necessary for long periods of uninterrupted flight and the release of energy substrates into the haemolymph. A range of neuropeptide receptors and neuropeptides modulate flight, fuel, feeding and metabolic homeostasis. Bottom: Flight muscle proteins are upregulated and muscle function protected by autophagy via TGF‐β signalling and mitophagy through JAK/STAT signalling. The JAK/STAT pathway further contributes to immune protection from pathogens and stress responses, along with a range of other factors that protect the migrant from oxidative damage. Metabolism and fuelling genes highlight the role of lipid storage, activation and mobilisation for migration and the role of key limiting enzymes of the glycolytic pathway for regulating flight performance. Asterisk indicates genes previously identified as associated with migration in monarch butterflies. The genes *foxo*, *fiz*, *EcR*, *Tdc1*, *Pdf*, *Akh*, *sNPF*, *dpp*, *put*, *Atg8a*, *Stat92E*, *CG4830* and CG17191 fall below 1.5 log2 fold change but within an adjusted *p*‐value of <.001 for differentially expressed genes. See discussion for details

We present a draft genome for the marmalade hoverfly *Episyrphys balteatus* and use this resource to identify differentially expressed genes between active migrants, caught as they traversed a high mountain pass in the Pyrenees during their migration southwards in the autumn, and nonmigratory summer individuals. Unlike previous analyses of insect migrants that utilised different migratory populations, demonstrating varying levels of migratory activity, we take advantage of seasonally differentiated forms that display radically different behaviour and physiology, and use differential gene expression analysis to identify genes with potential roles in migration. However, factors such as reproductive states, time of day, temperature, altitude, and photoperiod vary between the samples and may mean that a range of responses are also represented within the final data set (see Table S3). We order our discussion below based on categories of genes identified as most significantly up‐ or downregulated, or with the highest fold changes, from our analysis of the top up‐ and downregulated gene lists (Table 2; Table S6), together with the assignment of genes to enriched GO terms from the upregulated gene set (Figure 3; Tables S7 and S8). A summary of categories discussed in the following sections, together with gene expression levels is shown in Figure 4.

### 4.1 Environmental sensing, timing and navigation

Sensory perception of the environment is of great importance for initiation, maintenance and termination of migration, for circadian control, orientation and for locating food sources. We identified various genes with roles in sensory functions from our top upregulated list. These include *Rootletin* (*Root*) that encodes the major structural component of the ciliary rootlet and is involved in mechano‐ and chemosensation (Styczynska‐Soczka & Jarman, 2015), *Odorant‐binding protein 56a* (*Obp56a*) and *Neprilysin* 1 (*Nep1*). *Nep1* is involved in olfactory memory and neprilysins appear to function by turning off neuropeptide signals at the synapse or by the activation of neuromodulators such as *sNPF* (see below; Rose et al., 2009; Turrel et al., 2016). *Odorant‐binding proteins* (*OBPs*) have been identified as significantly overexpressed in *H. armigera* long‐distance fliers and subsequent analysis identified tissue specific expression of *OBP6* in the antennae, wings and thorax (Jones et al., 2015; Wang et al., 2020). Transgenic *D. melanogaster* overexpressing *H. armigera OBP6* in the flight muscle attain higher flight speeds, suggesting, together with the identification of potential binding affinity with triacylglycerols and phospholipids, that OBPs may also act as carriers of free fatty acids as part of the flight fuel pathway, a role worth further investigation in *Episyrphus* (Wang et al., 2020). Enrichment for the “system processes” GO term is also driven by many genes for sensory perception, particularly of light, smell and chemical stimulus and many of these genes are also shared across the “response to pheromone” GO term. This list includes *cryptochrome 1* (*cry1*, Figure S3) a circadian photoreceptor implicated as the input pathway for magnetic sensing and with a potential role in a magnetic inclination compass in monarch butterflies (Gegear et al., 2008; Wan et al., 2021). While the existence of equivalent magnetic compasses in migratory hoverflies remain to be investigated, *cry1* may play a role as a time giver for the time‐compensated sun compass as has been implicated in monarch butterflies (Sauman et al., 2005).

One of the most pleiotropic genes from our top upregulated list encodes the bZIP transcription factor: *vrille* (*vri*). *vri* is a clock‐controlled gene that acts as a repressor of the products of *Clk* and *cry* that control circadian functions (Blau & Young, 1999; Cyran et al., 2003; Glossop et al., 2003). In *D. melanogaster vri* mediates clock output to generate behavioural rhythms, including regulating levels of *pdf*, involved in the control of flight (see below; Blau & Young, 1999; Gunawardhana & Hardin, 2017). The promoter region of *vri* is bound by the circadian transcription factor Clock and by Foxo providing a link between the circadian system and Insulin/insulin‐like growth factor signalling (IIS, see next section; Bai et al., 2013; Shokri et al., 2019). *vri* may additionally interact with ecdysone and TGF‐β pathways (see below; Beckstead et al., 2005; Gauhar et al., 2009; George & Terracol, 1997). Partial loss of *vri* results in flight and other locomotory defects in *D. melanogaster* while Vri interacts with genes encoding actin‐binding proteins including *bent* (see below; Szuplewski et al., 2003). Finally, *vri* has previously been associated with directional flight in migratory monarch butterflies and is rhythmically expressed in the monarch brain (Lugena et al., 2019; Zhan et al., 2011; Zhu et al., 2009). Together, these observations suggest that *vri* may sit at a hub of molecular interactions in migrants, conceivably connecting environmental sensing and circadian control with IIS and downstream hormonal and transcriptional modulation of the migratory syndrome. Disentangling these interactions presents a considerable challenge.

### 4.2 Insulin/insulin‐like growth factor signalling

Insulin/insulin‐like growth factor signalling (IIS) forms an evolutionary conserved systemic nutritional and environmental sensor that regulates metabolism, growth, behaviour, life span and reproduction (Regan et al., 2020). For example, disruption of IIS in *D. melanogaster* shuts down reproduction and increases energy stores and stress tolerance mimicking a state of reproductive diapause (Baker & Thummel, 2007; Kops et al., 2002). IIS also appears to be central to diapause in other insects, and in mosquitos it has been shown to function upstream of JH (Sim & Denlinger, 2013). We detect strong down‐regulation of the *Insulin‐like receptor* (*InR*) from our top downregulated gene list. Upon binding of insulin‐like proteins, InR phosphorylates its substrate Chico resulting in downstream signalling events, with many of the diapause like‐states being mediated by the forkhead transcription factor Foxo. In the absence of IIS, Foxo is activated and translocated to the nucleus where it transcriptionally regulates many target genes (Martins et al., 2016). Mutants of *InR* in *D. melanogaster* show an increase in lifespan and are nonvitellogenic, conditions that can be reversed by treatment with a juvenile hormone (JH) analogue, demonstrating plausible mechanisms, through IIS repression and hormonal control, for the longevity and reproductive diapause seen in migratory hoverflies (Tatar et al., 2001). In addition, we detect strong downregulation of *Lnk*, an adaptor of InR that stabilises the interaction between the activated InR and Chico (Slack et al., 2010). Loss of *Lnk* function in *D. melanogaster* also results in increased lifespan and improved survival under conditions of oxidative stress and starvation as well as increasing carbohydrate and lipid levels (Slack et al., 2010). Further support for reduced IIS in migrants comes from upregulation of the Pi3K antagonist *Phosphatase and tensin homologue* (*Pten*), that reduces IIS activity (Goberdhan et al., 1999), and from the differential expression of known Foxo targets, including the positively regulated *vri* and *pudgy* (see below) that appear in our top‐up regulated list, and the negative regulation of targets such as *Lnk* and *dawdle* (see below; Bai et al., 2013; Goberdhan et al., 1999; Slack et al., 2010).

### 4.3 Hormonal control: juvenile hormone and ecdysone related genes

Hormonal control of migratory physiology is well documented in insect migrants with juvenile hormone (JH) playing a central role in the trade‐off between reproductive and migratory physiology (Rankin, 1991). In monarch butterflies for example, lower JH titres favour migration including reproductive diapause, increased longevity, and increased fat stores (Reppert et al., 2016). Top upregulated genes with roles in hormonal processes in *Episyrphus* migrants include *CG2680*, *CG11852* and two glucose‐methanol‐choline oxidoreductases: *CG9522* and *fezzik* (*fiz*, *CG9509*). *CG11852* encodes a protein with signatures of the Takeout superfamily and the haemolymph juvenile hormone binding proteins; suggesting a role in JH regulation (Xu et al., 2011). Likewise, *CG2680* is a Haloacid Dehalogenase (HAD) family member and expressed in the ring gland, the major endocrine organ of *D. melanogaster*, which includes the corpus allatum responsible for producing JH (Cao et al., 2009). Also of note is the downregulation of *juvenile hormone acid methyltransferase* (*jhamt*) encoding the enzyme responsible for the final step in the JH biosynthesis pathway and differentially expressed between summer and fall monarch butterflies (Green & Kronforst, 2019; Shinoda & Itoyama, 2003; Zhu et al., 2009).

Ecdysteroids also have an important role in regulating reproductive diapause with titres dropping at the onset of diapause and remaining low until its termination (Denlinger, 2002). *fiz* encodes an ecdysone oxidase involved in the degradation of ecdysone and in the regulation of growth rate and body size in *D. melanogaster* (Glaser‐Schmitt & Parsch, 2018; Iida et al., 2007; Takeuchi et al., 2005). A recent study has also implicated *fiz* in female starvation resistance when found in high expression variants of *D. melanogaster* (Glaser‐Schmitt et al., 2021). In addition, we identified downregulation of *Enhancer of bithorax* (*E[bx]*) from our top downregulated gene set, whose product in *D. melanogaster* binds the ecdysone receptor (*EcR*; also downregulated) in an ecdysone‐dependent manner to regulate the transcription of a large set of ecdysone‐responsive targets (Badenhorst et al., 2005), indicating that differential regulation of these targets may be important in migrant *Episyrphus* (see below).

### 4.4 Octopamine synthesis and migratory flight

In invertebrates, octopamine (OA) and tyramine (TA) play roles analogous to adrenaline and noradrenaline in vertebrates and regulate a wide variety of processes and metabolic pathways, including flight behaviour and physiology (Roeder, 2020). For example, *D. melanogaster* OA deficient flies decrease both flight duration and initiation, OA also acts on the fat body causing the release of energy substrates such as fatty acids and trehalose into the haemolymph (Brembs et al., 2007; Roeder, 2020). We find enrichment for the “phenol‐containing compound biosynthetic process” and “indole‐containing compound biosynthetic process” GO terms that are driven by enzymes involved in the synthesis of the biogenic amines TA, OA and dopamine. This includes the upregulation of *tyramine beta hydroxylase* (*tbh*) whose product converts tyramine into octopamine and is the key limiting enzyme in OA synthesis (Monastirioti et al., 1996). *tbh* has previously been associated with directional flight in migratory monarch butterflies (Zhu et al., 2009). Other factors associated with these pathways (but not the GO term) are also upregulated including *Tyrosine decarboxylase 1* (*Tdc1*) which is involved in the direct biosynthesis of tyramine and indirectly with the biosynthesis of octopamine; *nazgul* (*naz*) involved in TA catabolism and which causes reduced flight duration in *D. melanogaster* RNAi knockdown experiments (Ryglewski et al., 2017). Reviewing the roles of OA in metabolic traits, Roeder (2020) suggested that the “long periods of uninterrupted flight that are necessary to find new places and resources critically depend on high OA concentrations”, a view clearly supported for migratory flight in *Episyrphus* (Roeder, 2020). Finally, the heading‐direction network found in the central complex of the brain in monarch butterflies switches to code for a sun compass during steering flight and this process is mediated by OA (Beetz et al., 2022). Together these observations highlight the importance of OA in regulating long‐distance flight and potentially in initiating key navigational processes during *Episyrphus* migration.

### 4.5 Neuropeptide hormones

Insects utilise a diverse set of neuropeptide signalling molecules in a huge variety of neuron types to regulate physiology and behaviour (Nässel & Winther, 2010). We detected neuropeptide related genes enriched among the upregulated gene list for the “multicellular organismal process” GO term. These include a number of receptors: *Pigment‐dispersing factor receptor* (*Pdfr*) involved in modulating flight; *Adipokinetic hormone receptor* (*AkhR*) important for regulating circulating hemolymph carbohydrates and lipids; *short neuropeptide F receptor* (*sNPF‐R*) a factor with roles in feeding; foraging behaviour and appetitive memory and *Crustacean cardioactive peptide receptor* (*CCAP‐R*) which regulates feeding behaviour (Agrawal et al., 2013; Knapek et al., 2013; Williams et al., 2020). The neuropeptides associated with these receptors are also upregulated >1 fold but fall short of our stringent cut off. Other neuropeptides enriched under this GO term are *Corazonin* (*Crz*) and its receptor (*CrzR*), that are elevated during stress to coordinate increased food intake to regain metabolic homeostasis, indicating that these factors may play a role in the hyperphagy observed in captured *Episyrphus* migrants (Baggerman et al., 2002; Kubrak et al., 2016). The collection of enzymes, neuropeptide receptors and neuropeptides identified here are likely to play diverse roles in the migratory forms of *Episyrphus* and warrant further in‐depth investigation.

### 4.6 Muscle function: Genes involved in migratory flight

Muscle assembly is focused in the thorax of flies and is primary associated with flight. The flight muscles are separated into direct or indirect flight muscle (IFM) architecture. IFM is essential to flight, driving the up and downstroke of the wings through mechanisms in the wing hinge and through the contractions of the dorsal longitudinal muscles (DLM) and dorsal ventral muscles (DVM; Boettiger & Furshpan, 1952). We identified several genes with roles in flight muscle function, with *Lamin C* (*LamC*) topping the list of most significantly upregulated genes. Mutations in lamins cause a spectrum of diseases in humans, including muscular dystrophy and *LamC* has been shown to maintain proper function and morphology of tendon cells in *D. melanogaster*, which may be important in flight for transmitting muscular force to the exoskeleton (Uchino et al., 2013). Also in the top list is *Actin 88F* (*Act88F*), the only actin specifically found in IFM, where it plays a role in the highly specialised oscillatory contractions that power flight (Hiromi & Hotta, 1985).

Several other muscle genes important for flight appear in our GO enrichment analysis including several elastic proteins involved in flight muscle contraction encoded by the *sallimus* (*sls*) gene such as Projectin and Kettin (Bullard et al., 2006). *Kettin*, together with a second gene also identified from our enrichment analysis, *Collagen type IV alpha 1* (*Col4a1*), have been strongly associated with genomic signatures of selection related to migration in the monarch butterfly (Zhan et al., 2014). Whilst we detected upregulation of *col4a1*, it is the downregulation of this gene in migratory monarch populations that has been hypothesised to contribute to flight efficiency (Zhan et al., 2014). However, we note that the nonmigratory summer generation of the migratory population was used for this comparison, leaving an open question as to its expression levels in the migratory generation as assayed here. Other genes include *flightin* (*fln*) and *bent* (*bt*) that encode proteins associated with myosin thick filaments, contributing to the function of flight muscle (Bullard et al., 2006; Reedy et al., 2000). Finally, although just below our stringent cutoff, we also detect upregulation of *decapentaplegic* (*dpp*), a member of the TGF‐β superfamily (see next section) and a muscle‐secreted factor whose overexpression reduces foraging and feeding initiation (Robles‐Murguia et al., 2020), both behaviours suppressed during migratory flight in *Episyrphus*.

### 4.7 Transforming growth factor β signalling in migrants

The transforming growth factor β (TGF‐β) signalling pathway has two branches, initiated by different ligands: activins and bone morphogenetic proteins (BMPs) and is increasingly recognised as an important regulator of many physiological and metabolic processes, including of carbohydrate and lipid metabolism in response to stress and nutrient status (Chatterjee & Perrimon, 2021; Song et al., 2018; Upadhyay et al., 2017). One of the top downregulated genes in *Episyrphus* migrants is *Sno oncogene* (*Snoo*) encoding a protein that antagonises the product of *dpp* and facilitates activin signalling (Takaesu et al., 2006). Downregulation of *snoo* appears to reflect a general promotion of *dpp* signalling (including upregulation of *dpp* and *sog*) and repression of activin signalling (downregulation of *babo*, *put*, *daw*, and *plum*; *plum* is also in the top downregulated list) in migrants. This may be partially controlled by nutrient sensing through the IIS pathway and may provide beneficial consequences to behaviour and performance in migrants (Bai et al., 2013). For example, in *D. melanogaster*, reduced activin signalling occurs through Foxo repression of *daw* and the subsequent alleviation of translation repression of *Atg8a*, a key autophagy gene that leads to improvements in muscle performance and life span (Bai et al., 2013). Interestingly, variants in *Snoo* have also been uncovered in a screen for genetic modifiers of flight and BMP signalling may be important for modifying flight performance in natural populations (Spierer et al., 2021). The various roles of TGF‐β signalling in migrants clearly warrants further investigation.

### 4.8 JAK/STAT pathway and stress and immunity genes

Seasonal migration can profoundly affect interactions between hosts and pathogens, and modulation of immunity during this period may offset a greater exposure to infection experienced while moving through different geographic areas (Altizer et al., 2011). Activity of the downregulated gene *Enhancer of bithorax* (*E[bx]*) is required to maintain repression of Janus kinase/signal transducer and activator of transcription (JAK/STAT) pathway target genes in *D. melanogaster* (Kwon et al., 2008). The JAK/STAT pathway contributes to immune and stress responses and loss of *E(bx)* (as mirrored in migrants) leads to precocious activation of STAT target genes involved in immunity (Kwon et al., 2008; Myllymäki & Rämet, 2014). In support of this we find downregulation of the transmembrane receptor and transcription factor of the JAK/STAT pathway (*domeless* and *Stat92E*) and upregulation of immunity related targets for bacterial and viral infection (*vir‐1*, *Tep2*, *Tep3*) (Myllymäki & Rämet, 2014) suggesting a greater investment in immunity in *Episyrphus* migrants.

A consequence of energy‐demanding flight is a fast turnover of fuel and increased oxidative damage (Jenni‐Eiermann et al., 2014; Yan & Sohal, 2000). Within our top upregulated list we identified genes with potential roles in detoxification including *Cytochrome P450 4 s3* (*Cyp4s3*), *Glutathione transferase* (*GstD1*) and *α‐Esterase‐9* (*α‐Est9*). We also identified *multiple ankyrin repeats single KH domain* (*mask*) among the top downregulated genes in migrants. In *D. melanogaster mask* is expressed in flight muscle and may function as a novel negative regulator of mitophagy, counteracting damage sustained to mitochondria during flight (Katzemich et al., 2015; Zhu et al., 2015). Knockdown of *mask* in *D. melanogaster* Parkinson's disease models restores mitochondrial integrity and, in addition, Mask protein interacts with the JAK/STAT pathway through Dome (Fisher et al., 2018; Zhu et al., 2015). Several other genes involved in resistance to oxidative stress and longevity are included in our enriched GO terms including *Glial Lazarillo* (*GLaz*) whose overexpression in *D. melanogaster* increases resistance to oxidative stress, increases lifespan and resistance to starvation (Walker et al., 2006). The range of factors contributing to stress resistance highlight the likely importance of these protective measures for migratory activity.

### 4.9 Metabolism: Fuelling migratory flight

Insect flight may result in 100‐fold or greater increases in oxygen consumption over resting states (Arrese & Soulages, 2010). Fuelling for nonmigratory flight in species such as *D. melanogaster* is powered by glycogen, trehalose, and hexoses rather than fat (Sacktor & Hurlbut, 1966; Wigglesworth, 1949); in contrast, the fuelling of migratory flight in insects such as locusts utilises energy‐dense lipids to reduce the energetic cost of transporting extra weight. Reflecting this, in the top upregulated gene lists for migrants are two acyl‐CoA synthetases involved in activating fatty acids, allowing their participation in anabolic and catabolic pathways. These are a long‐chain fatty‐acid CoA ligase (*CG4830*) and *pudgy* (*pdgy*). In *D. melanogaster*, *pdgy* is a Foxo transcriptional target and mutants show increased sensitivity to starvation and lower fat stores, suggesting that these may be enhanced by *pdgy* up‐regulation in *Episyrphus* migrants (Thimgan et al., 2018; Xu et al., 2012). Other genes identified with roles in lipid metabolism include *Desaturase 1* (*Desat1*), with potential roles in fat storage and resistance to starvation and two neutral triglyceride lipases, *CG17191* and *CG17292*, both putatively involved in the conversion of stored triacylglycerol (TAG) to diacylglycerol (DAG), the main form of transported lipids (Horne et al., 2009; Parisi et al., 2013). Hence, these genes may play key roles in the storage and utilisation of fat to fuel migration. The potential importance of lipids as a fuel is further highlighted by the enrichment for the “fatty‐acyl‐CoA metabolic process” GO term, which includes a number of other long‐chain‐fatty‐acid‐CoA ligases and reductases. Finally, we detected downregulation of fat storage regulator *brummer* (*bmm*) that hydrolyses TAG to free fatty acids and DAG, and whose loss in *D. melanogaster* leads to increases in fat stores, mimicking the high lipid content observed in *Episyrphus* migrants (Grönke et al., 2005; Hondelmann & Poehling, 2007).

Carbohydrate metabolism genes also feature strongly within our migration‐associated gene set. Topping the upregulated gene list for carbohydrate metabolism is *Trehalose transporter 1–1* (*Tret1‐1*), responsible for the release of trehalose (the dominant haemolymph sugar) from the fat body and the incorporation of trehalose into other tissues (Kanamori et al., 2010). The most highly expressed of the carbohydrate metabolism genes are two alpha‐glucosidases: Glucosidase 2 α subunit (*GCS2α*; *CG14476*) and *Maltase A7* (*Mal‐A7*) which are most probably involved in the breakdown of starch into glucose (Inomata et al., 2019) and suggest an increased ability to process nutrients in migrants. *Hexokinase A* (*Hex‐A*) a gene that controls the entry of glucose into the glycolytic pathway was also highly expressed. *Hex‐A* is strongly expressed in *D. melanogaster* flight muscles and may function near capacity during flight, as flight performance is sensitive to variation in its activity (Eanes et al., 2006). Enrichment for the “carbohydrate metabolic process” GO term in migrants is driven by a large set of genes involved in carbohydrate metabolism including another classic regulatory enzyme that functions near capacity during flight: *Glycogen phosphorylase* (*GlyP*), which is activated by *Adipokinetic hormone* (Eanes et al., 2006). Other genes include *Glycerol‐3‐phosphate dehydrogenase 1* (*Gpdh*) whose variation in *D. melanogaster* populations is known to affect power output during flight, and *phosphoenolpyruvate carboxykinase 1* (*Pepck1*) that catalyses the rate‐limiting step in gluconeogenesis (Barnes & Laurie‐Ahlberg, 1986; Hanson & Patel, 2006).

We also detected genes involved in protein metabolic processes (*CG8132* and *CG6428*) in our top upregulated gene lists suggesting that amino acids may form part of the fuel mix for flight, as seen in some Hymenoptera and in tsetse flies (Bursell, 1963; Teulier et al., 2016). Finally, we identified several up‐regulated genes involved in energy metabolism including *Malic enzyme* (*Men*), which has been shown to control both energy metabolism and cellular ROS levels, and its overexpression in *D. melanogaster* results in increased life span (Kim et al., 2015; Paik et al., 2012).

## 5 CONCLUSIONS

Pre‐empting deteriorating seasonal conditions in northern Europe, *Episyrphus* hoverflies undergo radical changes to their behaviour and physiology, allowing them to undertake long‐distance southwards migration. We show here that these changes are underpinned by large modifications in gene regulation, with over 1500 genes showing strong evidence of differential expression between migrants and nonmigrants. Interrogation of this gene set reveals a remarkable range of potential roles for these genes in metabolism, muscle structure and function, hormonal regulation, immunity, stress resistance, flight and feeding behaviour, longevity, reproductive diapause and sensory perception. These features of the migrant phenotype have probably arisen by the integration and modification of established pathways, for example IIS for diapause and longevity, JAK/SAT for immunity, or through the enhancement of existing capabilities, for example of flight by the modulation of pathways leading to OA production and improvements to the storage, mobilisation and utilisation of fuel. Understanding how this collection of traits evolved together to create an integrated migratory syndrome remains to be elucidated. However, this study provides a theoretical framework for the genetic basis of the migratory syndrome (Figure 4), depicting the changes as a cascade from environmental sensing, through hormonal control to downstream migratory traits and identifies key genes associated with each step. This framework may be used to direct future studies into the extent to which evolution of migration has converged or diverged among hoverflies and other insects. Importantly, several of the genes and pathways identified here have previously been associated with migration in Lepidoptera, or with migration‐associated traits in other species, suggesting the existence of a “migratory gene package” and a common genetic basis (of at least some features and across some lineages) for migration (Liedvogel et al., 2011). Finally, while we have uncovered a comprehensive picture of migration‐associated genes expressed during active migration, further work remains to uncover the full spatial and temporal details of gene regulation and to explore the role of these genes and pathways using post‐genomic functional approaches. In this regard, the high‐quality draft genome and expression data generated in this study is a significant resource to accelerate the development of hoverflies as an important model for migration research.

## AUTHOR CONTRIBUTIONS

Toby Doyle, Eva Jimenez‐Guri and Karl R. Wotton designed and performed research, analysed data and wrote the manuscript. Will L. S. Hawkes and Richard Massey contributed to sample collection. Federica Mantica, Jon Permanyer and Manuel Irimia performed research and analysed data. Luca Cozzuto, Toni Hermoso Pulido, Tobias Baril and Alex Hayward analysed the data. Jason W. Chapman and Chris Bass contributed to research design.

## CONFLICT OF INTEREST

The authors have declared no conflict of interest for this article.

## Supporting information


File S1

File S2

## Data Availability

The datasets and metadata supporting the results of this article have been deposited at DDBJ/ENA/GenBank and are available via Bioproject PRJNA720810. The genome assembly described in this paper has been deposited under the accession JAGTYB000000000. The version described in this paper is version JAGTYB010000000. Tissue level RNAseq data is available from the Gene Expression Omnibus (GEO) repository via GSE205498. Genome and repeat annotation files are available on figshare 10.6084/m9.figshare.19333181.
