# Supplementary material for: Genome‐wide transcriptomic changes reveal the genetic pathways involved in insect migration
Source: Mol Ecol. 2022 Jul 12;31(16):4332–50. doi: 10.1111/mec.16588 (PMC9546057; doi:10.1111/mec.16588)
Supplement: Supplementary file 1 — File S1 [file MEC-31-4332-s002.docx]

**Supplementary File for Doyle et al.** Genome-wide transcriptomic changes reveal the genetic pathways central to insect migratory flight.

**Table S1.** *Episyrphus* genome report

| Assembly | Ebal_assembly_scaffold_fasta |
| --- | --- |
| # contigs (>= 0 bp) | 1231 |
| # contigs (>= 1000 bp) | 1231 |
| Total length (>= 0 bp) | 565681355 |
| Total length (>= 1000 bp) | 565681355 |
| # contigs | 1231 |
| Largest contig | 13960754 |
| Total length | 565681355 |
| GC (%) | 31.22 |
| N50 | 2544270 |
| N75 | 1149455 |
| L50 | 63 |
| L75 | 145 |
| # N's per 100 kbp | 0.10 |

**Table S2.** BUSCO scores for the *Episyrphus* genome against a Dipteran BUSCO gene set. BUSCO scores based on the diptera_odb10 BUSCO set using v4.1.2.

| BUSCO genome score | C:93.7% [S:90.6%, D:3.1%], F:2.3%, M:4.0%, n:3285 |
| --- | --- |
| Complete BUSCOs (C) | 3079 |
| Complete and single-copy BUSCOs (S) | 2976 |
| Complete and duplicated BUSCOs (D) | 103 |
| Fragmented BUSCOs (F) | 74 |
| Missing BUSCOs (M) | 132 |
| Total BUSCO groups searched | 3285 |

**Table S3.** *Episyrphus balteatus* sample collection data

| **ID** | **Location** | **Migratory status** | **Date collected** | **Time of collection** | **Temp (°C)** | **L:D regime (hours.minutes)** | **Altitude (metres)** |
| --- | --- | --- | --- | --- | --- | --- | --- |
| 1nm | Penryn | non-migrant | 13.07.2018 | 10:09 | 20.5 | 16.02:07.58 | 91 |
| 2nm | Penryn | non-migrant | 13.07.2018 | 08:34 | 18.7 | 16.02:7.58 | 91 |
| 4nm | Penryn | non-migrant | 16.07.2018 | 13:58 | 21.6 | 15.56:8.4 | 91 |
| 5nm | Penryn | non-migrant | 16.07.2018 | 14:30 | 22.3 | 15.56:8.4 | 91 |
| 8nm | Penryn | non-migrant | 17.07.2018 | 09:15 | 19.7 | 15.54:8.6 | 91 |
| 9nm | Penryn | non-migrant | 17.07.2018 | 09:11 | 19.3 | 15.54:8.6 | 91 |
| 12nm | Penryn | non-migrant | 17.07.2018 | 09:29 | 19 | 15.54:8.6 | 91 |
| 18nm | Penryn | non-migrant | 18.07.2018 | 08:54 | 20 | 15.51:8.9 | 91 |
| 13nm | Penryn | non-migrant | 17.07.2018 | 11:38 | 21.3 | 15.54:8.6 | 91 |
| 15nm | Penryn | non-migrant | 17.07.2018 | 14:10 | 20.3 | 15.54:8.6 | 91 |
| 16nm | Penryn | non-migrant | 17.07.2018 | 14:04 | 20.4 | 15.54:8.6 | 91 |
| 17nm | Penryn | non-migrant | 18.07.2018 | 08:54 | 20 | 15.51:8.9 | 91 |
| 10nm | Penryn | non-migrant | 17.07.2018 | 09:39 | 17 | 15.54:8.6 | 91 |
| 11nm | Penryn | non-migrant | 17.07.2018 | 09:22 | 19.4 | 15.54:8.6 | 91 |
| 14nm | Penryn | non-migrant | 17.07.2018 | 11:13 | 20.8 | 15.54:8.6 | 91 |
| 8m | Bujaruelo | migrant | 4.10.2018 | 13:52 | 12.3 | 11.36:12.24 | 2270 |
| 9m | Bujaruelo | migrant | 4.10.2018 | 14:16 | 12.6 | 11.36:12.24 | 2270 |
| 15m | Bujaruelo | migrant | 21.09.2018 | 14:20 | 15.7 | 12.13:11.47 | 2270 |
| 24m | Bujaruelo | migrant | 4.10.2018 | 15:15 | 12.4 | 11.36:12.24 | 2270 |
| 25m | Bujaruelo | migrant | 4.10.2018 | 16:32 | 11.8 | 11.36:12.24 | 2270 |
| 1m | Bujaruelo | migrant | 4.10.2018 | 15.27 | 12.4 | 11.36:12.24 | 2270 |
| 18m | Bujaruelo | migrant | 4.10.2018 | 15:11 | 12.4 | 11.36:12.24 | 2270 |
| 16m | Bujaruelo | migrant | 4.10.2018 | 14.59 | 12.6 | 11.36:12.24 | 2270 |
| 7m | Bujaruelo | migrant | 21.09.2018 | 11:24 | 14.2 | 12.13:11.47 | 2270 |
| 37m | Bujaruelo | migrant | 4.10.2018 | 15.37 | 12.4 | 11.36:12.24 | 2270 |
| 3m | Bujaruelo | migrant | 4.10.2018 | 16.31 | 11.8 | 11.36:12.24 | 2270 |
| 6m | Bujaruelo | migrant | 4.10.2018 | 15.29 | 12.4 | 11.36:12.24 | 2270 |
| 20m | Bujaruelo | migrant | 4.10.2018 | 15.37 | 12.4 | 11.36:12.24 | 2270 |
| 29m | Bujaruelo | migrant | 4.10.2018 | 16:40 | 11.8 | 11.36:12.24 | 2270 |
| 33m | Bujaruelo | migrant | 4.10.2018 | 15:15 | 12.4 | 11.36:12.24 | 2270 |

**Table S4.** Primers for qPCR

| Type | Gene symbol and name | Primer Fwd (5'-3') | Primer Rev (5'-3') | Efficiency |
| --- | --- | --- | --- | --- |
| Control | RPS20: Ribosomal protein S20 | ATCGAAAAGCCCCAAGGAGG | GGATCAAGTCACGGCAGACA | 1.01 |
| Control | RpL32: Ribosomal protein L32 | ATTCGTCATCAGTCGGACCG | GAAACGACGACGGACTCTGT | 0.99 |
| Target | α-Est9: α-Esterase-9 | TTTTCGGTGATAGCGCTGGT | CCCGTATAACCAGCCTCCAC | 0.96 |
| Target | pdgy: pudgy | TTCGTGATTCCTGTGCTGCT | TGTAACCGTTAAGCCCGCTT | 0.98 |
| Target | jv: javelin | CGAACGAATGCCCGCATAAG | GTGCCTGTGTTGTTGTCGTC | 0.95 |
| Target | Sp7: Serine protease 7 | GCTGGTTGGGGTAGAACTCT | TGTCCACCCACACATAGCTG | 1 |
| Target | α-Est7: α-Esterase-7 | TTGGGGGTCGGTGAAACAAT | TACGGCTCAACAGTTGGTCC | 0.95 |
| Target | Bruce: BIR repeat containing ubiquitin-conjugating enzyme | ATGCCCGACATAGCCACAAT | TCAGAGAGAATGTGTGGCGG | 1.05 |
| Target | Ubr3: Ubr3 ubiquitin ligase | GACCTACGCCCGATTTGGAT | ACCACCGGCTATCAACCAAC | 0.94 |
| Target | RhoGEF2: Rho guanine nucleotide exchange factor 2 | TGTACCCGTTTCCGACTTGG | CTTCTGAGGACCAGCACCAG | 0.97 |
| Target | PlexB: Plexin B | GCACACCAACATCAACAGCC | GGACCGGTTACAGCTTGTGT | 0.95 |
| Target | unknown potential enok: enoki mushroom | TGACAATGTCCCGAAGCAGT | CAACAGCTGCAAAACCCCAA | 0.96 |

**Table S5** qPCR expression differences of selected genes

| gene | group 1 | group 2 | n1 | n2 | stat | df | p | p.adj | p.adj.signif |
| --- | --- | --- | --- | --- | --- | --- | --- | --- | --- |
| alphaest7 | m | nm | 5 | 5 | 1.938687 | 4.065238 | 1.23E-01 | 0.369 | ns |
| alphaest9 | m | nm | 5 | 5 | 11.78948 | 7.974303 | 2.52E-06 | 2.52E-05 | **** |
| bruce | m | nm | 5 | 5 | 2.641236 | 7.006967 | 3.33E-02 | 0.1332 | ns |
| Ubr3 (cg42593) | m | nm | 5 | 5 | -0.15026 | 6.660643 | 8.85E-01 | 0.885 | ns |
| enok | m | nm | 5 | 5 | 3.604461 | 7.83328 | 7.19E-03 | 0.03595 | * |
| jv | m | nm | 5 | 5 | 17.42455 | 5.060905 | 1.03E-05 | 9.27E-05 | **** |
| pdgy | m | nm | 5 | 5 | 7.845573 | 5.1091 | 4.89E-04 | 0.003423 | ** |
| plexb | m | nm | 5 | 5 | -5.59938 | 5.545687 | 1.79E-03 | 0.01074 | * |
| rhogef2 | m | nm | 5 | 5 | 1.102723 | 7.238322 | 3.05E-01 | 0.61 | ns |
| sp7 | m | nm | 5 | 5 | 6.7563 | 6.503364 | 3.64E-04 | 0.002912 | ** |


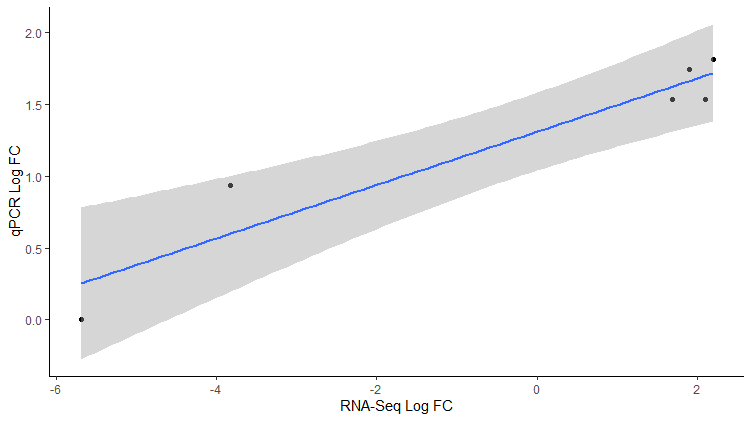


**Figure S1.** Validation of RNA-seq by qPCR. Individual qPCR fold-changes plotted against RNA-seq values. Only genes that exhibited significant expression between migratory and non-migratory phenotypes were included in this analysis. Fold-changes are log_2_-transformed. A simple linear regression line is presented (with 95% CIs). We detected a strong correlation between observed fold changes between the RNA-seq and qPCR data (cor = 0.9509202, t = 6.1461, df = 4, p-value = 0.003554)

**Figure S2.** Gene expression plotted as Z-scores across all samples for the most differentially expressed genes (DEGs) from non-migrant and migrant *Episyrphus*. The Z-score gives the number of standard-deviations that a gene expression is away from the mean of all the values for each gene. Migrants and non-migrant samples cluster together and changes in gene expression are consistent across these groups.

**Figure S3** Maximum likelihood phylogenetic analysis of cryptochromes reveals that the differentially expressed *Episyrphus* gene found on scaffold 38 is a *Drosophila*-like type 1 cryptochrome. The phylogenetic analysis was carried out with NGPhylogeny.fr (see Lemoine *et al.* NGPhylogeny.fr: new generation phylogenetic services for non-specialists. *Nucleic Acids Research* 2019).

**Table S6**. Top down-regulated genes ordered by adjust p-value (padj) and log2fold expression differences

| Gene ID | Gene Symbol | Fold | padj | Potential roles |
| --- | --- | --- | --- | --- |
| *Top by padj* |  |  |  |  |
| EBAG0006250 | Bruce | -41.9 | 2.48E-216 | Blocks apoptosis |
| EBAG0009539 | detonator CG14446 | -9.9 | 8.07E-179 | Reproduction |
| EBAG0000157 | mask | -15.1 | 3.66E-169 | Mitophagy |
| EBAG0008051 | RhoGEF2 | -13.3 | 1.50E-168 | actin organization |
| EBAG0003084 | hyd | -34.1 | 2.46E-160 | Wnt signalling |
| EBAG0011394 | CG42593 | -18.6 | 3.43E-154 | Ubiquitination |
| EBAG0010211 | Wnk | -17.6 | 1.30E-135 | Wnt signalling |
| EBAG0003852 | PlexB | -30.7 | 1.29E-133 | Fat storage |
| EBAG0007070 | dikar | -14.2 | 2.64E-129 | Memory |
| EBAG0010736 | EBAG0010736 | -14.2 | 7.18E-127 |  |
| EBAG0002458 | Hers | -8.9 | 1.18E-123 | Epigenetic gene silencing |
| EBAG0005152 | Snoo | -9.0 | 1.27E-110 | TGF-β through BMP branch |
| EBAG0009074 | amon | -7.8 | 1.22E-105 | Processing of neuropeptides |
| EBAG0002722 | E(bx) | -35.4 | 1.55E-99 | Hormonal regulation / JAK/STAT |
| EBAG0000374 | InR | -6.6 | 1.34E-92 | IIS |
| EBAG0010552 | fra | -13.5 | 1.65E-90 | Axon guidance |
| EBAG0000500 | plum | -9.5 | 4.30E-89 | TGF-β through activin branch |
| EBAG0006329 | CG14435 | -9.2 | 1.84E-87 | unknown |
| EBAG0003114 | alpha-Man-IIb | -10.7 | 4.27E-86 | Encapsulation response |
| EBAG0002209 | CaMKII | -4.3 | 7.02E-85 | Ca[2+]-calmodulin-regulated serine/threonine protein kinase |
| *Top by fold change* | |  |  |  |
| EBAG2000491 | Vm26Aa | -131.6 | 7.90E-63 | Egg structure |
| EBAG0010980 | Cp36 | -103.0 | 4.84E-51 | Egg structure |
| EBAG0010981 | Cp38 | -93.7 | 3.97E-53 | Egg structure |
| EBAG0009034 | CG14309 | -85.7 | 1.33E-42 | Egg structure |
| EBAG0006191 | Cpr30B | -72.3 | 7.06E-33 | Cuticle structure |
| EBAG0010978 | Cp7Fa | -54.9 | 1.47E-37 | Egg structure |
| EBAG0003194 | Fcp3C | -53.6 | 3.39E-33 | Egg structure |
| EBAG0003289 | CG43778 (hbt) | -47.6 | 5.37E-70 | Sensory perception |
| EBAG0000675 | ndl | -43.4 | 4.70E-41 | Egg structure |
| EBAG0007021 | yellow-g | -42.6 | 4.59E-29 | Egg structure |
| EBAG0006250 | Bruce | -41.9 | 2.48E-216 | Blocks apoptosis |
| EBAG2000603 | CG14187 | -36.5 | 1.88E-20 | Egg structure |
| EBAG0002722 | E(bx) | -35.4 | 1.55E-99 | Hormonal regulation / JAK/STAT |
| EBAG0007197 | CG12716 | -34.7 | 6.05E-21 | Egg structure |
| EBAG0003084 | hyd | -34.1 | 2.46E-160 | Wnt signalling |
| EBAG0002526 | Obp19c | -33.7 | 2.44E-32 | Sensory perception |
| EBAG2001405 | zen | -31.1 | 3.16E-25 | Oogenesis |
| EBAG0003852 | PlexB | -30.7 | 1.29E-133 | Lipid accumulation |
| EBAG0000488 | CG11448 | -25.4 | 4.02E-39 | lysosome distribution |
| EBAG0004658 | Hsp26 | -24.8 | 1.73E-19 | Ovarian HSP |

**Table S7. Processes GO term enrichment for up-regulated *Episyrphus* migration-associated genes.** Enrichment calculated using GOrilla against all Episyrphus transcripts as the background. 'P-value' is the enrichment p-value computed according to the mHG or HG model. This p-value is not corrected for multiple testing of GO terms. 'FDR q-value' is the correction of the above p-value for multiple testing using the Benjamini and Hochberg (1995) method. Namely, for the ith term (ranked according to p-value) the FDR q-value is (p-value * number of GO terms) / i. Enrichment (N, B, n, b) is defined as follows: N - is the total number of genes; B - is the total number of genes associated with a specific GO term; n - is the number of genes in the top of the user's input list or in the target set when appropriate; b - is the number of genes in the intersection. Enrichment = (b/n) / (B/N).

| **GO Term** | **Description** | **P-value** | **FDR q-value** | **Enrichment** |
| --- | --- | --- | --- | --- |
| GO:0007600 | sensory perception | 1.37E-08 | 9.89E-05 | 2.68 |
| GO:0003008 | system process | 5.06E-08 | 1.83E-04 | 1.99 |
| GO:0006030 | chitin metabolic process | 8.24E-08 | 1.98E-04 | 3.91 |
| GO:1901071 | glucosamine-containing compound metabolic process | 5.97E-07 | 1.08E-03 | 3.51 |
| GO:0030239 | myofibril assembly | 7.06E-07 | 1.02E-03 | 7.79 |
| GO:0006040 | amino sugar metabolic process | 8.04E-07 | 9.67E-04 | 3.45 |
| GO:0007608 | sensory perception of smell | 1.35E-06 | 1.39E-03 | 3.81 |
| GO:0019236 | response to pheromone | 1.95E-06 | 1.75E-03 | 7.14 |
| GO:0006022 | aminoglycan metabolic process | 2.68E-06 | 2.15E-03 | 3.11 |
| GO:0007606 | sensory perception of chemical stimulus | 3.48E-06 | 2.51E-03 | 2.8 |
| GO:0006936 | muscle contraction | 9.95E-06 | 6.53E-03 | 6.12 |
| GO:0003012 | muscle system process | 1.14E-05 | 6.86E-03 | 5.36 |
| GO:0040003 | chitin-based cuticle development | 1.52E-05 | 8.46E-03 | 2.52 |
| GO:0017144 | drug metabolic process | 2.77E-05 | 1.43E-02 | 2.07 |
| GO:0042335 | cuticle development | 5.73E-05 | 2.76E-02 | 2.34 |
| GO:0050877 | nervous system process | 7.77E-05 | 3.50E-02 | 1.74 |
| GO:0032501 | multicellular organismal process | 8.10E-05 | 3.44E-02 | 1.34 |
| GO:0045214 | sarcomere organization | 1.81E-04 | 7.26E-02 | 3.69 |
| GO:0031032 | actomyosin structure organization | 2.43E-04 | 9.24E-02 | 2.72 |
| GO:0006012 | galactose metabolic process | 3.48E-04 | 1.26E-01 | 8.57 |
| GO:0010927 | cellular component assembly involved in morphogenesis | 3.70E-04 | 1.27E-01 | 3.71 |
| GO:0007498 | mesoderm development | 4.01E-04 | 1.31E-01 | 2.99 |
| GO:0046189 | phenol-containing compound biosynthetic process | 6.39E-04 | 2.00E-01 | 5.95 |
| GO:0005975 | carbohydrate metabolic process | 7.18E-04 | 2.16E-01 | 1.93 |
| GO:0044281 | small molecule metabolic process | 7.54E-04 | 2.18E-01 | 1.45 |
| GO:0035337 | fatty-acyl-CoA metabolic process | 7.59E-04 | 2.10E-01 | 4.17 |
| GO:0033499 | galactose catabolic process via UDP-galactose | 8.10E-04 | 2.16E-01 | 10.71 |
| GO:0019388 | galactose catabolic process | 8.10E-04 | 2.09E-01 | 10.71 |
| GO:0042435 | indole-containing compound biosynthetic process | 8.10E-04 | 2.01E-01 | 10.71 |
| GO:0019752 | carboxylic acid metabolic process | 9.98E-04 | 2.40E-01 | 1.66 |

**Table S8. Processes GO term enrichment for down-regulated *Episyrphus* migration-associated genes.** Terms as in Table S6.

| **GO Term** | **Description** | **P-value** | **FDR q-value** | **Enrichment** |
| --- | --- | --- | --- | --- |
| GO:0022402 | cell cycle process | 1.94E-24 | 1.40E-20 | 2.87 |
| GO:0050789 | regulation of biological process | 1.99E-21 | 7.19E-18 | 1.43 |
| GO:0050794 | regulation of cellular process | 2.38E-21 | 5.71E-18 | 1.46 |
| GO:0022414 | reproductive process | 2.46E-21 | 4.43E-18 | 2.06 |
| GO:1903047 | mitotic cell cycle process | 2.86E-20 | 4.13E-17 | 3.37 |
| GO:0065007 | biological regulation | 7.56E-20 | 9.09E-17 | 1.37 |
| GO:0060255 | regulation of macromolecule metabolic process | 3.67E-19 | 3.78E-16 | 1.66 |
| GO:0022412 | cellular process involved in reproduction in multicellular organism | 4.33E-18 | 3.90E-15 | 2.26 |
| GO:0006259 | DNA metabolic process | 2.63E-17 | 2.11E-14 | 3.14 |
| GO:0019222 | regulation of metabolic process | 3.72E-17 | 2.68E-14 | 1.58 |
| GO:0051726 | regulation of cell cycle | 1.02E-16 | 6.71E-14 | 2.94 |
| GO:0051171 | regulation of nitrogen compound metabolic process | 2.80E-16 | 1.68E-13 | 1.62 |
| GO:2000112 | regulation of cellular macromolecule biosynthetic process | 3.44E-16 | 1.91E-13 | 1.79 |
| GO:0010556 | regulation of macromolecule biosynthetic process | 5.87E-16 | 3.02E-13 | 1.78 |
| GO:0080090 | regulation of primary metabolic process | 1.17E-15 | 5.60E-13 | 1.61 |
| GO:0010468 | regulation of gene expression | 2.24E-15 | 1.01E-12 | 1.69 |
| GO:0031326 | regulation of cellular biosynthetic process | 5.85E-15 | 2.48E-12 | 1.73 |
| GO:0009889 | regulation of biosynthetic process | 7.15E-15 | 2.86E-12 | 1.73 |
| GO:0031323 | regulation of cellular metabolic process | 2.31E-14 | 8.77E-12 | 1.56 |
| GO:0032502 | developmental process | 3.00E-14 | 1.08E-11 | 1.52 |
| GO:0006260 | DNA replication | 4.31E-14 | 1.48E-11 | 5.29 |
| GO:0048519 | negative regulation of biological process | 7.20E-14 | 2.36E-11 | 1.67 |
| GO:0007346 | regulation of mitotic cell cycle | 9.80E-14 | 3.07E-11 | 3.3 |
| GO:0051276 | chromosome organization | 2.71E-13 | 8.13E-11 | 3.12 |
| GO:0071840 | cellular component organization or biogenesis | 3.67E-13 | 1.06E-10 | 1.47 |
| GO:0048523 | negative regulation of cellular process | 4.61E-13 | 1.28E-10 | 1.71 |
| GO:0003006 | developmental process involved in reproduction | 4.76E-13 | 1.27E-10 | 2.07 |
| GO:0006270 | DNA replication initiation | 4.96E-13 | 1.28E-10 | 7.37 |
| GO:0019219 | regulation of nucleobase-containing compound metabolic process | 5.76E-13 | 1.43E-10 | 1.67 |
| GO:0051128 | regulation of cellular component organization | 6.87E-13 | 1.65E-10 | 1.86 |
| GO:0006974 | cellular response to DNA damage stimulus | 1.04E-12 | 2.42E-10 | 3.02 |
| GO:0016043 | cellular component organization | 1.34E-12 | 3.03E-10 | 1.47 |
| GO:1903046 | meiotic cell cycle process | 1.55E-12 | 3.39E-10 | 3.17 |
| GO:0007051 | spindle organization | 2.53E-12 | 5.36E-10 | 3.89 |
| GO:0007052 | mitotic spindle organization | 5.73E-12 | 1.18E-09 | 5.22 |
| GO:0000724 | double-strand break repair via homologous recombination | 1.06E-11 | 2.13E-09 | 5.1 |
| GO:0000725 | recombinational repair | 1.06E-11 | 2.07E-09 | 5.1 |
| GO:0006310 | DNA recombination | 1.10E-11 | 2.09E-09 | 4.39 |
| GO:0090304 | nucleic acid metabolic process | 1.52E-11 | 2.81E-09 | 1.74 |
| GO:0010629 | negative regulation of gene expression | 2.60E-11 | 4.69E-09 | 2.1 |
| GO:0048856 | anatomical structure development | 5.43E-11 | 9.56E-09 | 1.58 |
| GO:0010564 | regulation of cell cycle process | 6.28E-11 | 1.08E-08 | 2.96 |
| GO:0006355 | regulation of transcription, DNA-templated | 8.96E-11 | 1.50E-08 | 1.67 |
| GO:2001141 | regulation of RNA biosynthetic process | 8.96E-11 | 1.47E-08 | 1.67 |
| GO:1903506 | regulation of nucleic acid-templated transcription | 8.96E-11 | 1.44E-08 | 1.67 |
| GO:0000280 | nuclear division | 1.02E-10 | 1.59E-08 | 4.67 |
| GO:0000727 | double-strand break repair via break-induced replication | 1.05E-10 | 1.61E-08 | 9.14 |
| GO:0006281 | DNA repair | 1.17E-10 | 1.75E-08 | 3.05 |
| GO:0009892 | negative regulation of metabolic process | 1.21E-10 | 1.78E-08 | 1.86 |
| GO:0010605 | negative regulation of macromolecule metabolic process | 1.39E-10 | 2.01E-08 | 1.89 |
| GO:0065004 | protein-DNA complex assembly | 1.70E-10 | 2.41E-08 | 4.57 |
| GO:0140013 | meiotic nuclear division | 1.72E-10 | 2.39E-08 | 4.76 |
| GO:0051252 | regulation of RNA metabolic process | 2.04E-10 | 2.77E-08 | 1.61 |
| GO:0007059 | chromosome segregation | 2.32E-10 | 3.09E-08 | 3.93 |
| GO:0010558 | negative regulation of macromolecule biosynthetic process | 2.80E-10 | 3.67E-08 | 2.21 |
| GO:2000113 | negative regulation of cellular macromolecule biosynthetic process | 2.80E-10 | 3.61E-08 | 2.21 |
| GO:0048522 | positive regulation of cellular process | 3.44E-10 | 4.36E-08 | 1.54 |
| GO:0048518 | positive regulation of biological process | 4.68E-10 | 5.83E-08 | 1.5 |
| GO:0006996 | organelle organization | 8.59E-10 | 1.05E-07 | 1.64 |
| GO:0071103 | DNA conformation change | 1.76E-09 | 2.11E-07 | 3.86 |
| GO:0044260 | cellular macromolecule metabolic process | 2.12E-09 | 2.51E-07 | 1.45 |
| GO:0009890 | negative regulation of biosynthetic process | 2.18E-09 | 2.54E-07 | 2.11 |
| GO:0031327 | negative regulation of cellular biosynthetic process | 2.18E-09 | 2.50E-07 | 2.11 |
| GO:0007049 | cell cycle | 2.25E-09 | 2.53E-07 | 2.79 |
| GO:1902850 | microtubule cytoskeleton organization involved in mitosis | 4.08E-09 | 4.53E-07 | 3.99 |
| GO:0050793 | regulation of developmental process | 5.07E-09 | 5.54E-07 | 1.73 |
| GO:0006302 | double-strand break repair | 5.38E-09 | 5.79E-07 | 3.68 |
| GO:0033043 | regulation of organelle organization | 6.60E-09 | 7.00E-07 | 2.03 |
| GO:0006261 | DNA-dependent DNA replication | 6.97E-09 | 7.29E-07 | 4.84 |
| GO:0048869 | cellular developmental process | 8.25E-09 | 8.50E-07 | 1.61 |
| GO:0048285 | organelle fission | 9.01E-09 | 9.15E-07 | 3.85 |
| GO:0031324 | negative regulation of cellular metabolic process | 9.93E-09 | 9.95E-07 | 1.84 |
| GO:0060284 | regulation of cell development | 1.03E-08 | 1.02E-06 | 2.11 |
| GO:0051960 | regulation of nervous system development | 1.07E-08 | 1.05E-06 | 2.07 |
| GO:0000226 | microtubule cytoskeleton organization | 1.10E-08 | 1.06E-06 | 2.41 |
| GO:0022607 | cellular component assembly | 1.15E-08 | 1.09E-06 | 1.71 |
| GO:0098813 | nuclear chromosome segregation | 1.64E-08 | 1.54E-06 | 4.21 |
| GO:0050767 | regulation of neurogenesis | 1.97E-08 | 1.83E-06 | 2.27 |
| GO:0007010 | cytoskeleton organization | 2.69E-08 | 2.46E-06 | 1.99 |
| GO:0030261 | chromosome condensation | 3.31E-08 | 2.98E-06 | 4.46 |
| GO:0045595 | regulation of cell differentiation | 3.41E-08 | 3.03E-06 | 1.96 |
| GO:0007143 | female meiotic nuclear division | 4.12E-08 | 3.63E-06 | 5.63 |
| GO:0051172 | negative regulation of nitrogen compound metabolic process | 4.85E-08 | 4.22E-06 | 1.84 |
| GO:0051783 | regulation of nuclear division | 5.14E-08 | 4.41E-06 | 3.67 |
| GO:0006323 | DNA packaging | 5.32E-08 | 4.52E-06 | 4.34 |
| GO:0006468 | protein phosphorylation | 6.05E-08 | 5.08E-06 | 2.29 |
| GO:0000278 | mitotic cell cycle | 6.35E-08 | 5.27E-06 | 2.65 |
| GO:0071824 | protein-DNA complex subunit organization | 6.93E-08 | 5.68E-06 | 3.19 |
| GO:0007281 | germ cell development | 6.98E-08 | 5.66E-06 | 2.33 |
| GO:0007276 | gamete generation | 8.34E-08 | 6.69E-06 | 2.27 |
| GO:0010721 | negative regulation of cell development | 1.05E-07 | 8.31E-06 | 3.43 |
| GO:2000026 | regulation of multicellular organismal development | 1.20E-07 | 9.40E-06 | 1.75 |
| GO:0007088 | regulation of mitotic nuclear division | 1.38E-07 | 1.07E-05 | 3.62 |
| GO:0032501 | multicellular organismal process | 1.56E-07 | 1.20E-05 | 1.44 |
| GO:0048468 | cell development | 1.74E-07 | 1.32E-05 | 1.88 |
| GO:0045132 | meiotic chromosome segregation | 2.37E-07 | 1.78E-05 | 5.44 |
| GO:0048477 | oogenesis | 2.51E-07 | 1.87E-05 | 2.47 |
| GO:0007292 | female gamete generation | 3.02E-07 | 2.23E-05 | 2.46 |
| GO:0007275 | multicellular organism development | 3.90E-07 | 2.85E-05 | 2.55 |
| GO:0051303 | establishment of chromosome localization | 4.83E-07 | 3.49E-05 | 6.9 |
| GO:0006725 | cellular aromatic compound metabolic process | 5.68E-07 | 4.06E-05 | 1.44 |
| GO:0000075 | cell cycle checkpoint | 6.44E-07 | 4.56E-05 | 3.77 |
| GO:0016310 | phosphorylation | 6.49E-07 | 4.54E-05 | 2.01 |
| GO:0050768 | negative regulation of neurogenesis | 7.16E-07 | 4.97E-05 | 3.44 |
| GO:1902299 | pre-replicative complex assembly involved in cell cycle DNA replication | 7.27E-07 | 4.99E-05 | 8.72 |
| GO:0036388 | pre-replicative complex assembly | 7.27E-07 | 4.95E-05 | 8.72 |
| GO:0006267 | pre-replicative complex assembly involved in nuclear cell cycle DNA replication | 7.27E-07 | 4.90E-05 | 8.72 |
| GO:0006139 | nucleobase-containing compound metabolic process | 7.40E-07 | 4.94E-05 | 1.46 |
| GO:0017148 | negative regulation of translation | 8.25E-07 | 5.46E-05 | 3.89 |
| GO:0033554 | cellular response to stress | 8.67E-07 | 5.68E-05 | 1.81 |
| GO:1901360 | organic cyclic compound metabolic process | 1.05E-06 | 6.82E-05 | 1.42 |
| GO:0007389 | pattern specification process | 1.07E-06 | 6.86E-05 | 1.85 |
| GO:0045664 | regulation of neuron differentiation | 1.15E-06 | 7.32E-05 | 2.3 |
| GO:0050000 | chromosome localization | 1.23E-06 | 7.80E-05 | 6.41 |
| GO:0006401 | RNA catabolic process | 1.28E-06 | 8.00E-05 | 3.22 |
| GO:0045934 | negative regulation of nucleobase-containing compound metabolic process | 1.30E-06 | 8.11E-05 | 1.92 |
| GO:0051225 | spindle assembly | 1.54E-06 | 9.48E-05 | 3.93 |
| GO:0045596 | negative regulation of cell differentiation | 1.83E-06 | 1.12E-04 | 2.74 |
| GO:0007304 | chorion-containing eggshell formation | 2.01E-06 | 1.22E-04 | 5.54 |
| GO:0046483 | heterocycle metabolic process | 2.14E-06 | 1.28E-04 | 1.43 |
| GO:0006325 | chromatin organization | 2.57E-06 | 1.53E-04 | 1.97 |
| GO:0090306 | spindle assembly involved in meiosis | 2.81E-06 | 1.66E-04 | 5.98 |
| GO:0051239 | regulation of multicellular organismal process | 2.90E-06 | 1.70E-04 | 1.58 |
| GO:0048513 | animal organ development | 3.07E-06 | 1.78E-04 | 1.68 |
| GO:0010604 | positive regulation of macromolecule metabolic process | 3.35E-06 | 1.93E-04 | 1.57 |
| GO:0009987 | cellular process | 3.44E-06 | 1.97E-04 | 1.12 |
| GO:0051253 | negative regulation of RNA metabolic process | 3.56E-06 | 2.02E-04 | 1.92 |
| GO:0001558 | regulation of cell growth | 3.82E-06 | 2.15E-04 | 3.02 |
| GO:0030703 | eggshell formation | 3.86E-06 | 2.16E-04 | 5.25 |
| GO:0051173 | positive regulation of nitrogen compound metabolic process | 4.59E-06 | 2.55E-04 | 1.57 |
| GO:0006950 | response to stress | 5.22E-06 | 2.88E-04 | 1.5 |
| GO:0007017 | microtubule-based process | 5.28E-06 | 2.89E-04 | 1.83 |
| GO:0018107 | peptidyl-threonine phosphorylation | 5.85E-06 | 3.17E-04 | 5.61 |
| GO:0018210 | peptidyl-threonine modification | 5.85E-06 | 3.15E-04 | 5.61 |
| GO:0048609 | multicellular organismal reproductive process | 5.89E-06 | 3.15E-04 | 1.86 |
| GO:0033044 | regulation of chromosome organization | 6.02E-06 | 3.19E-04 | 2.43 |
| GO:0030704 | vitelline membrane formation | 6.40E-06 | 3.37E-04 | 8.54 |
| GO:0007080 | mitotic metaphase plate congression | 6.40E-06 | 3.35E-04 | 8.54 |
| GO:0007305 | vitelline membrane formation involved in chorion-containing eggshell formation | 6.40E-06 | 3.32E-04 | 8.54 |
| GO:0035803 | egg coat formation | 6.40E-06 | 3.30E-04 | 8.54 |
| GO:0006357 | regulation of transcription by RNA polymerase II | 6.64E-06 | 3.40E-04 | 1.53 |
| GO:1902679 | negative regulation of RNA biosynthetic process | 6.71E-06 | 3.41E-04 | 1.92 |
| GO:0045892 | negative regulation of transcription, DNA-templated | 6.71E-06 | 3.39E-04 | 1.92 |
| GO:1903507 | negative regulation of nucleic acid-templated transcription | 6.71E-06 | 3.36E-04 | 1.92 |
| GO:0034249 | negative regulation of cellular amide metabolic process | 6.75E-06 | 3.36E-04 | 3.39 |
| GO:0048583 | regulation of response to stimulus | 7.40E-06 | 3.65E-04 | 1.48 |
| GO:0045786 | negative regulation of cell cycle | 8.82E-06 | 4.33E-04 | 2.79 |
| GO:0006268 | DNA unwinding involved in DNA replication | 9.09E-06 | 4.43E-04 | 6.98 |
| GO:0051310 | metaphase plate congression | 9.09E-06 | 4.40E-04 | 6.98 |
| GO:0045665 | negative regulation of neuron differentiation | 9.80E-06 | 4.71E-04 | 4.12 |
| GO:1902975 | mitotic DNA replication initiation | 1.00E-05 | 4.79E-04 | 9.97 |
| GO:1902315 | nuclear cell cycle DNA replication initiation | 1.00E-05 | 4.76E-04 | 9.97 |
| GO:1902292 | cell cycle DNA replication initiation | 1.00E-05 | 4.73E-04 | 9.97 |
| GO:0051255 | spindle midzone assembly | 1.00E-05 | 4.70E-04 | 9.97 |
| GO:0044786 | cell cycle DNA replication | 1.22E-05 | 5.68E-04 | 4.75 |
| GO:0007166 | cell surface receptor signaling pathway | 1.26E-05 | 5.84E-04 | 1.92 |
| GO:0051961 | negative regulation of nervous system development | 1.75E-05 | 8.04E-04 | 2.55 |
| GO:0040007 | growth | 1.81E-05 | 8.26E-04 | 2.19 |
| GO:0048589 | developmental growth | 1.81E-05 | 8.21E-04 | 2.19 |
| GO:0061982 | meiosis I cell cycle process | 1.98E-05 | 8.94E-04 | 3.03 |
| GO:0009893 | positive regulation of metabolic process | 2.07E-05 | 9.29E-04 | 1.48 |
| GO:0048638 | regulation of developmental growth | 2.16E-05 | 9.60E-04 | 1.99 |
| GO:0071897 | DNA biosynthetic process | 2.21E-05 | 9.76E-04 | 3.86 |
| GO:0016321 | female meiosis chromosome segregation | 2.28E-05 | 1.00E-03 | 6.34 |
| GO:0085029 | extracellular matrix assembly | 2.34E-05 | 1.02E-03 | 7.48 |
| GO:0051983 | regulation of chromosome segregation | 2.41E-05 | 1.05E-03 | 3.6 |
| GO:0040008 | regulation of growth | 2.89E-05 | 1.25E-03 | 1.83 |
| GO:0051301 | cell division | 3.17E-05 | 1.36E-03 | 2.31 |
| GO:0006402 | mRNA catabolic process | 3.75E-05 | 1.60E-03 | 3.01 |
| GO:0000212 | meiotic spindle organization | 4.15E-05 | 1.76E-03 | 3.92 |
| GO:0051716 | cellular response to stimulus | 4.23E-05 | 1.78E-03 | 1.58 |
| GO:0022604 | regulation of cell morphogenesis | 4.34E-05 | 1.82E-03 | 2.2 |
| GO:0022603 | regulation of anatomical structure morphogenesis | 4.54E-05 | 1.89E-03 | 1.86 |
| GO:0048646 | anatomical structure formation involved in morphogenesis | 4.86E-05 | 2.02E-03 | 1.77 |
| GO:0043170 | macromolecule metabolic process | 5.35E-05 | 2.21E-03 | 1.22 |
| GO:0071526 | semaphorin-plexin signaling pathway | 5.52E-05 | 2.26E-03 | 8.31 |
| GO:0031570 | DNA integrity checkpoint | 6.08E-05 | 2.48E-03 | 3.78 |
| GO:0042023 | DNA endoreduplication | 6.10E-05 | 2.47E-03 | 4.98 |
| GO:0010975 | regulation of neuron projection development | 6.33E-05 | 2.55E-03 | 2.36 |
| GO:0006271 | DNA strand elongation involved in DNA replication | 6.43E-05 | 2.58E-03 | 6.64 |
| GO:0022616 | DNA strand elongation | 6.43E-05 | 2.56E-03 | 6.64 |
| GO:0016572 | histone phosphorylation | 6.43E-05 | 2.55E-03 | 6.64 |
| GO:0007127 | meiosis I | 6.43E-05 | 2.53E-03 | 6.64 |
| GO:0007056 | spindle assembly involved in female meiosis | 6.43E-05 | 2.52E-03 | 6.64 |
| GO:0070192 | chromosome organization involved in meiotic cell cycle | 6.46E-05 | 2.52E-03 | 3.52 |
| GO:0033045 | regulation of sister chromatid segregation | 6.46E-05 | 2.50E-03 | 3.52 |
| GO:0007093 | mitotic cell cycle checkpoint | 6.46E-05 | 2.49E-03 | 3.52 |
| GO:0007169 | transmembrane receptor protein tyrosine kinase signaling pathway | 6.66E-05 | 2.55E-03 | 2.41 |
| GO:0006417 | regulation of translation | 7.03E-05 | 2.68E-03 | 2.3 |
| GO:0045787 | positive regulation of cell cycle | 7.89E-05 | 3.00E-03 | 2.85 |
| GO:0045935 | positive regulation of nucleobase-containing compound metabolic process | 8.50E-05 | 3.21E-03 | 1.64 |
| GO:0006275 | regulation of DNA replication | 8.75E-05 | 3.29E-03 | 3.65 |
| GO:0033047 | regulation of mitotic sister chromatid segregation | 8.75E-05 | 3.27E-03 | 3.65 |
| GO:0007379 | segment specification | 8.75E-05 | 3.25E-03 | 3.65 |
| GO:0051246 | regulation of protein metabolic process | 8.85E-05 | 3.27E-03 | 1.56 |
| GO:0031325 | positive regulation of cellular metabolic process | 9.31E-05 | 3.43E-03 | 1.46 |
| GO:0000076 | DNA replication checkpoint | 9.91E-05 | 3.63E-03 | 5.37 |
| GO:0051094 | positive regulation of developmental process | 1.00E-04 | 3.65E-03 | 1.79 |
| GO:0040016 | embryonic cleavage | 1.01E-04 | 3.64E-03 | 9.97 |
| GO:0051382 | kinetochore assembly | 1.01E-04 | 3.63E-03 | 9.97 |
| GO:0009303 | rRNA transcription | 1.01E-04 | 3.61E-03 | 9.97 |
| GO:0010977 | negative regulation of neuron projection development | 1.05E-04 | 3.75E-03 | 4.69 |
| GO:0010608 | posttranscriptional regulation of gene expression | 1.06E-04 | 3.75E-03 | 2.06 |
| GO:0006464 | cellular protein modification process | 1.10E-04 | 3.89E-03 | 1.42 |
| GO:0036211 | protein modification process | 1.10E-04 | 3.88E-03 | 1.42 |
| GO:0048749 | compound eye development | 1.19E-04 | 4.15E-03 | 2.38 |
| GO:0007423 | sensory organ development | 1.23E-04 | 4.28E-03 | 1.99 |
| GO:0007167 | enzyme linked receptor protein signaling pathway | 1.40E-04 | 4.86E-03 | 2.17 |
| GO:0000956 | nuclear-transcribed mRNA catabolic process | 1.72E-04 | 5.95E-03 | 2.91 |
| GO:0006277 | DNA amplification | 1.73E-04 | 5.93E-03 | 4.43 |
| GO:0051383 | kinetochore organization | 1.77E-04 | 6.06E-03 | 7.12 |
| GO:0051270 | regulation of cellular component movement | 1.83E-04 | 6.22E-03 | 2.26 |
| GO:0016072 | rRNA metabolic process | 1.87E-04 | 6.34E-03 | 2.13 |
| GO:0051052 | regulation of DNA metabolic process | 1.93E-04 | 6.52E-03 | 2.66 |
| GO:0009880 | embryonic pattern specification | 1.95E-04 | 6.54E-03 | 2.57 |
| GO:0009966 | regulation of signal transduction | 2.07E-04 | 6.91E-03 | 1.45 |
| GO:0030111 | regulation of Wnt signaling pathway | 2.10E-04 | 6.99E-03 | 2.35 |
| GO:0006928 | movement of cell or subcellular component | 2.16E-04 | 7.15E-03 | 1.49 |
| GO:0120035 | regulation of plasma membrane bounded cell projection organization | 2.22E-04 | 7.30E-03 | 2.07 |
| GO:0016458 | gene silencing | 2.22E-04 | 7.28E-03 | 2.4 |
| GO:0032268 | regulation of cellular protein metabolic process | 2.27E-04 | 7.41E-03 | 1.54 |
| GO:0051129 | negative regulation of cellular component organization | 2.28E-04 | 7.40E-03 | 2.01 |
| GO:1901990 | regulation of mitotic cell cycle phase transition | 2.31E-04 | 7.49E-03 | 2.46 |
| GO:0045931 | positive regulation of mitotic cell cycle | 2.34E-04 | 7.53E-03 | 3.32 |
| GO:0097485 | neuron projection guidance | 2.36E-04 | 7.55E-03 | 1.81 |
| GO:0001654 | eye development | 2.50E-04 | 7.97E-03 | 2.21 |
| GO:0008150 | biological_process | 2.53E-04 | 8.05E-03 | 1.04 |
| GO:0031344 | regulation of cell projection organization | 2.53E-04 | 8.02E-03 | 2.06 |
| GO:0016570 | histone modification | 2.57E-04 | 8.09E-03 | 2.02 |
| GO:0016569 | covalent chromatin modification | 2.57E-04 | 8.06E-03 | 2.02 |
| GO:0010557 | positive regulation of macromolecule biosynthetic process | 2.70E-04 | 8.43E-03 | 1.59 |
| GO:0032508 | DNA duplex unwinding | 2.72E-04 | 8.46E-03 | 4.2 |
| GO:0030308 | negative regulation of cell growth | 2.72E-04 | 8.43E-03 | 4.2 |
| GO:1901987 | regulation of cell cycle phase transition | 2.78E-04 | 8.57E-03 | 2.42 |
| GO:0030334 | regulation of cell migration | 2.87E-04 | 8.83E-03 | 2.49 |
| GO:0009653 | anatomical structure morphogenesis | 2.90E-04 | 8.88E-03 | 1.4 |
| GO:0034655 | nucleobase-containing compound catabolic process | 3.06E-04 | 9.30E-03 | 2.14 |
| GO:2000145 | regulation of cell motility | 3.47E-04 | 1.05E-02 | 2.46 |
| GO:0045930 | negative regulation of mitotic cell cycle | 3.47E-04 | 1.05E-02 | 2.74 |
| GO:1903827 | regulation of cellular protein localization | 3.47E-04 | 1.04E-02 | 2.74 |
| GO:0070925 | organelle assembly | 3.54E-04 | 1.06E-02 | 1.88 |
| GO:0030707 | ovarian follicle cell development | 3.56E-04 | 1.06E-02 | 2.53 |
| GO:1901991 | negative regulation of mitotic cell cycle phase transition | 3.74E-04 | 1.11E-02 | 2.99 |
| GO:1901988 | negative regulation of cell cycle phase transition | 3.74E-04 | 1.11E-02 | 2.99 |
| GO:0043412 | macromolecule modification | 3.74E-04 | 1.10E-02 | 1.36 |
| GO:0030516 | regulation of axon extension | 4.14E-04 | 1.21E-02 | 3.99 |
| GO:0061387 | regulation of extent of cell growth | 4.14E-04 | 1.21E-02 | 3.99 |
| GO:0009792 | embryo development ending in birth or egg hatching | 4.18E-04 | 1.22E-02 | 3.13 |
| GO:0034248 | regulation of cellular amide metabolic process | 4.24E-04 | 1.23E-02 | 1.96 |
| GO:0040012 | regulation of locomotion | 4.30E-04 | 1.24E-02 | 2.18 |
| GO:0002066 | columnar/cuboidal epithelial cell development | 4.31E-04 | 1.24E-02 | 2.49 |
| GO:0008608 | attachment of spindle microtubules to kinetochore | 4.33E-04 | 1.24E-02 | 6.23 |
| GO:0016577 | histone demethylation | 4.33E-04 | 1.24E-02 | 6.23 |
| GO:0070076 | histone lysine demethylation | 4.33E-04 | 1.23E-02 | 6.23 |
| GO:0051231 | spindle elongation | 4.33E-04 | 1.23E-02 | 6.23 |
| GO:0010389 | regulation of G2/M transition of mitotic cell cycle | 4.54E-04 | 1.28E-02 | 3.59 |
| GO:1902749 | regulation of cell cycle G2/M phase transition | 4.54E-04 | 1.28E-02 | 3.59 |
| GO:0000290 | deadenylation-dependent decapping of nuclear-transcribed mRNA | 4.62E-04 | 1.29E-02 | 7.97 |
| GO:0030517 | negative regulation of axon extension | 4.62E-04 | 1.29E-02 | 7.97 |
| GO:0070193 | synaptonemal complex organization | 4.62E-04 | 1.28E-02 | 7.97 |
| GO:0007411 | axon guidance | 4.67E-04 | 1.29E-02 | 1.78 |
| GO:0051254 | positive regulation of RNA metabolic process | 5.23E-04 | 1.44E-02 | 1.58 |
| GO:0034622 | cellular protein-containing complex assembly | 5.28E-04 | 1.45E-02 | 1.65 |
| GO:0050770 | regulation of axonogenesis | 5.34E-04 | 1.46E-02 | 2.63 |
| GO:0051784 | negative regulation of nuclear division | 5.42E-04 | 1.48E-02 | 4.98 |
| GO:2001251 | negative regulation of chromosome organization | 5.49E-04 | 1.49E-02 | 3.05 |
| GO:0032392 | DNA geometric change | 6.11E-04 | 1.65E-02 | 3.8 |
| GO:0006334 | nucleosome assembly | 6.11E-04 | 1.64E-02 | 3.8 |
| GO:0031345 | negative regulation of cell projection organization | 6.11E-04 | 1.64E-02 | 3.8 |
| GO:0002064 | epithelial cell development | 6.24E-04 | 1.67E-02 | 2.42 |
| GO:0009891 | positive regulation of biosynthetic process | 6.71E-04 | 1.79E-02 | 1.52 |
| GO:0031328 | positive regulation of cellular biosynthetic process | 6.71E-04 | 1.78E-02 | 1.52 |
| GO:0051240 | positive regulation of multicellular organismal process | 7.40E-04 | 1.95E-02 | 1.67 |
| GO:0010628 | positive regulation of gene expression | 7.57E-04 | 1.99E-02 | 1.5 |
| GO:0007422 | peripheral nervous system development | 8.02E-04 | 2.10E-02 | 3.11 |
| GO:0032879 | regulation of localization | 8.50E-04 | 2.22E-02 | 1.49 |
| GO:0048813 | dendrite morphogenesis | 8.51E-04 | 2.22E-02 | 2.12 |
| GO:0030717 | oocyte karyosome formation | 8.76E-04 | 2.27E-02 | 3.62 |
| GO:0061988 | karyosome formation | 8.76E-04 | 2.27E-02 | 3.62 |
| GO:0007140 | male meiotic nuclear division | 8.76E-04 | 2.26E-02 | 3.62 |
| GO:0034502 | protein localization to chromosome | 8.95E-04 | 2.30E-02 | 5.54 |
| GO:0006482 | protein demethylation | 8.95E-04 | 2.29E-02 | 5.54 |
| GO:0008214 | protein dealkylation | 8.95E-04 | 2.28E-02 | 5.54 |
| GO:0009794 | regulation of mitotic cell cycle, embryonic | 8.95E-04 | 2.27E-02 | 5.54 |
| GO:0110118 | negative regulation of compound eye photoreceptor cell differentiation | 8.95E-04 | 2.26E-02 | 5.54 |
| GO:0051093 | negative regulation of developmental process | 9.14E-04 | 2.31E-02 | 1.79 |
| GO:0044818 | mitotic G2/M transition checkpoint | 9.22E-04 | 2.32E-02 | 4.6 |
| GO:0046011 | regulation of oskar mRNA translation | 9.22E-04 | 2.31E-02 | 4.6 |
| GO:0008356 | asymmetric cell division | 9.72E-04 | 2.43E-02 | 2.26 |
| GO:0060828 | regulation of canonical Wnt signaling pathway | 9.72E-04 | 2.42E-02 | 2.26 |
